# QuickConc: A Rapid, Efficient, and Power‐Free eDNA Concentration Method With Cationic‐Assisted Capture

**DOI:** 10.1002/ece3.72269

**Published:** 2025-10-08

**Authors:** Tomohiro Kuroita, Qianqian Wu, Ryo Iwamoto, Toshifumi Minamoto

**Affiliations:** ^1^ AdvanSentinel Inc. Osaka Japan; ^2^ Shionogi & Co., Ltd. Osaka Japan; ^3^ Graduate School of Human Development and Environment Kobe University Kobe Hyogo Japan

**Keywords:** environmental DNA (eDNA), filtration, MiFish, nucleic acid concentration, quantitative real‐time PCR, water sampling

## Abstract

Environmental DNA (eDNA) analysis is effective for non‐invasive biodiversity monitoring, as it reveals species distribution and abundance without ecosystem disruption. Four key steps in eDNA analysis include water preservation, DNA capture, DNA extraction, and detection. Among these, the capture of eDNA has attracted significant research interest due to the variability of water samples. Although various methods for eDNA concentration have been developed, including filtration using disc or cartridge filters and passive samplers, no single method is universally applicable because of the variabilities of eDNA distribution and water characteristics, including turbidity levels. Therefore, the development of alternative eDNA concentration methods is important for advancing eDNA research. This study introduces QuickConc, a novel nucleic acid capture method that combines an enhancer of benzalkonium chloride with dispersed silica glass fibers, allowing better binding between nucleic acids and glass fibers. Our results indicate that this approach enhances eDNA capture and extraction efficiency by likely improving the interaction between glass fibers and eDNA. We tested QuickConc in three environments using qPCR and metabarcoding. QuickConc yielded 1.3–3 times more total eDNA compared to glass fiber filtration and Sterivex methods under our experimental settings. Species‐specific qPCR results showed that QuickConc detected 2–10 times higher copy numbers compared to the other two methods. Metabarcoding analyses using the MiFish method revealed that the number of fish species detected in river water was higher with QuickConc compared to other methods, while in sea water, the number of fish species detected was similar to the glass fiber filtration and Sterivex methods. QuickConc offers new options for eDNA analysis for biodiversity monitoring and conservation strategies.

## Graphical abstract

Environmental DNA (eDNA) analysis allows non‐invasive monitoring of biodiversity, but effective eDNA capture remains challenging due to variable water characteristics. This study introduces QuickConc, a novel method combining benzalkonium chloride and silica glass fibers, which improved eDNA binding, capture, and extraction efficiency across river, sea, and pond environments. QuickConc offers new options for eDNA analysis for biodiversity monitoring and conservation strategies.

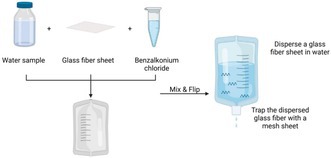

## 1 Introduction

Developing minimally invasive and less destructive biomonitoring techniques is crucial for biodiversity conservation (Kerry et al. 2022; Margules and Pressey 2000) and effective management policies (Jackson et al. 2001; Jungmeier and Yenilmez Arpa 2022). Environmental DNA (eDNA) analysis has become a powerful and non‐invasive tool for ecological monitoring. It originates from various biological sources and is present in dissolved, particle‐bound, or encapsulated form (Barnes and Turner 2016; Foote et al. 2012; Mauvisseau et al. 2022; Power et al. 2023; Rees et al. 2014). By analyzing water samples using quantitative polymerase chain reaction (qPCR) or metabarcoding with next‐generation sequencing (NGS), eDNA analysis provides valuable insights into species distribution and abundance (Civade et al. 2016; Jackman et al. 2021; Robson et al. 2016; Taberlet et al. 2018). This technique has been widely adopted in aquatic ecosystem surveys, with ongoing global standardization efforts (Shu et al. 2020).

Environmental DNA detectability is significantly affected by four key steps: water preservation, DNA capture, DNA extraction, and detection. The main eDNA capture methods are filtration (Allison et al. 2021; Bairoliya et al. 2022; Muha et al. 2019) and passive eDNA sampling (Kirtane et al. 2020; Verdier et al. 2022). Traditional laboratory‐based filtration methods, which concentrate tens of milliliters to several liters of water (Tsuji et al. 2019), can be limited by turbidity, time consumption, or equipment requirements (Bessey et al. 2021; Liu et al. 2024; Ruan et al. 2022; Sato et al. 2017). On‐site techniques such as syringe‐based Sterivex filtration (Miya et al. 2016), gravity filtration (Gold et al. 2021; Oka et al. 2022), or electric samplers (Thomas et al. 2018) offer practical alternatives but may still face clogging or operational constraints in highly turbid waters. Passive sampling methods (Bessey et al. 2021; Kirtane et al. 2020; Rivera et al. 2021; Verdier et al. 2022; Mariani et al. 2019) allow for simple, long‐term monitoring but have limited temporal resolution, and they complicate quantitative interpretation.

The choice of filter pore size influences the size fractions of eDNA captured (Liu et al. 2024; Majaneva et al. 2018; Power et al. 2023; Rivera et al. 2021; Zhao et al. 2021). Suzuki et al. (2023) recently developed a concentration technique using dispersed glass fibers to capture eDNA, followed by gravity filtration. This approach leverages DNA's affinity for silica (the main component of glass). The method is unique because it uses filters with relatively large pores (180 μm), compared to those traditionally used (glass fiber filters: 0.7–3.0 μm and Sterivex: 0.22–0.45 μm) (Minamoto et al. 2021; Takahashi et al. 2023). It combines eDNA with glass fibers in environmental water prior to filtration. This technique leverages the binding affinity of DNA for glass fibers, using larger pore‐sized filters and potentially mitigating clogging issues. Suzuki et al. (2023) primarily focused on on‐site detection; however, their simplified nucleic acid extraction method leaves room for improvement in terms of eDNA recovery efficiency and inhibitor removal.

The affinity between silica and DNA is well‐known to rely on both electrostatic (phosphate–silanol) and hydrophobic interactions (Shi et al. 2015; Vandeventer et al. 2012). Chaotropic agents, such as guanidine thiocyanate, are commonly used to facilitate this binding. Benzalkonium chloride (BAC), a quaternary ammonium compound and common disinfectant, has also been studied as an eDNA preservative at higher concentrations (Takahara et al. 2020); however, its ability to enhance eDNA binding specifically to dispersed glass fibers has not yet been tested extensively. We hypothesized that BAC, with its positive charge and hydrophobic tail, could form a molecular bridge between the negatively charged DNA and silica, thereby boosting DNA capture.

In this study, we developed QuickConc, a novel DNA capture method using dispersed glass fibers with BAC. First, we evaluated whether BAC improves total eDNA and species‐specific eDNA yields across three filtration methods: QuickConc, glass fiber filters, and Sterivex cartridges (Experiment 1). Next, we compared QuickConc against glass fiber filters and Sterivex cartridges across three types of aquatic environments (river, sea, pond) for both total DNA yield and species‐specific qPCR (Experiment 2). Finally, we used metabarcoding (MiFish) to evaluate how QuickConc affects fish community composition estimates in river and sea water. Our findings show that QuickConc can increase eDNA yields and, in some contexts, improve species detection, at least among the methods compared in this study. This offers a new tool for eDNA research and biomonitoring.

## 2 Materials and Methods

**FIGURE 1:**
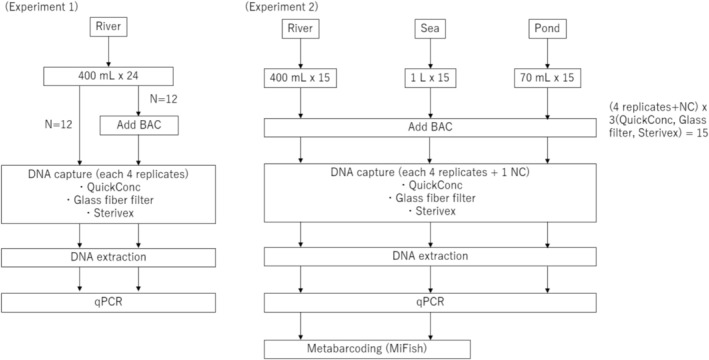
Experiment design diagram. Volumes of 400 mL, 1 L, and 70 mL per sample were collected from river, sea, and pond waters, respectively. In the experiment depicted in Experiment 1, four replicates per condition were collected, and each was subjected to DNA extraction. For the experiments presented in Experiment 2, four replicates and one negative control sample per method were collected. The DNA extracts were subjected to metabarcoding and qPCR analysis. NC, Negative control.

We conducted two experiments: Experiment 1 evaluated the effect of BAC addition across three filtration methods, while Experiment 2 compared QuickConc with conventional methods across three types of aquatic environments. Figure 1 provides an overview of the experiments' designs.

### 2.1 Experiment 1: Evaluation of BAC Addition Across Three Methods

#### 2.1.1 Sample Collection for Experiment 1

Surface water samples (400 mL each) were collected from the Kanzaki River (34°44′02.8″N 135°27′34.3″E) on March 5, 2025, and again on June 3–4, 2025, for the preliminary evaluation of optimal BAC concentration and extraction buffer conditions. Four replicates were collected. Each sample was immediately transferred to the laboratory. The non‐BAC sample was processed without modification, while BAC was added to samples in the BAC group to a final concentration of 0.001% w/v (see left‐hand diagram in Figure 1).

#### 2.1.2 Concentration and DNA Extraction for Experiment 1

**FIGURE 2:**
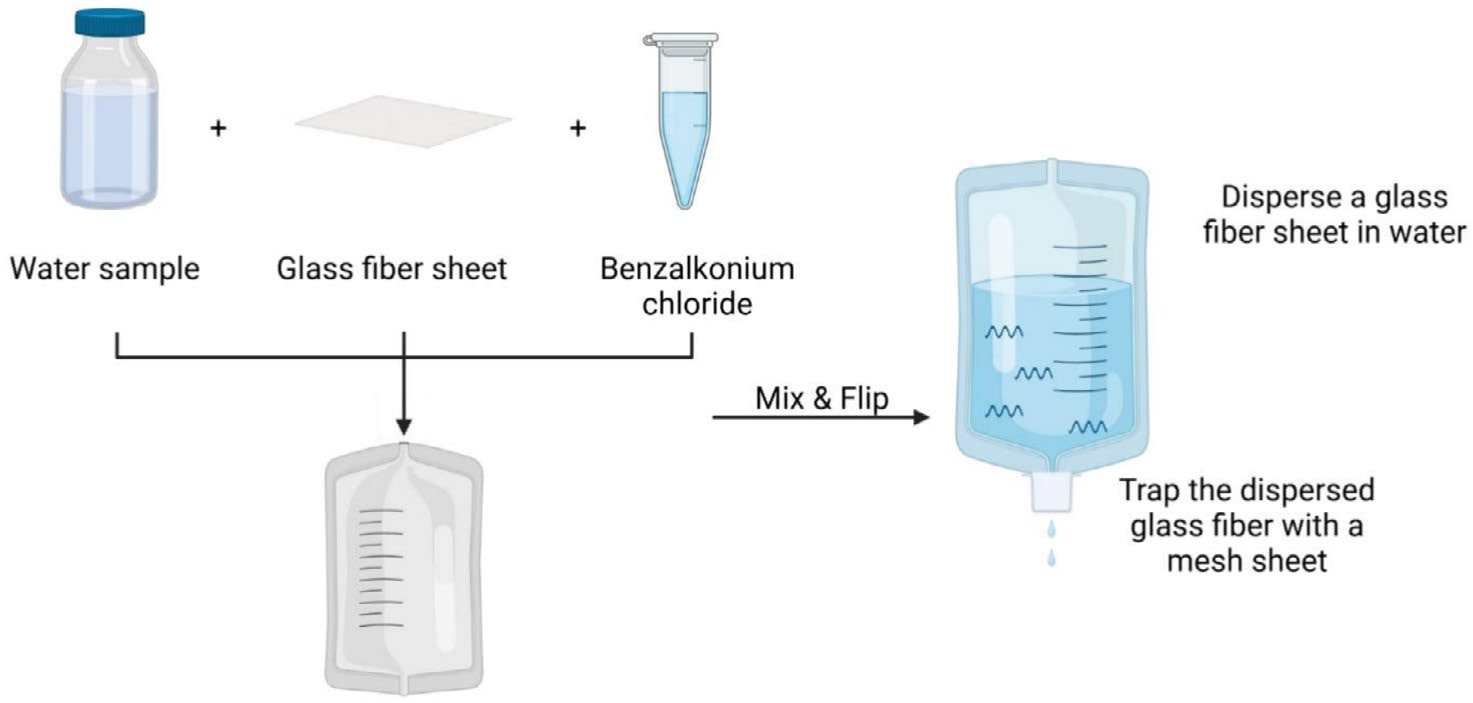
Workflow of the QuickConc method for nucleic acid concentration from environmental water samples. This figure illustrates the workflow of the newly developed QuickConc method, a streamlined protocol for the concentration of eDNA from environmental water samples. This approach involves the addition of a glass fiber sheet and benzalkonium chloride to the environmental water, followed by mixing. The subsequent filtration through a mesh filter efficiently captures the glass fiber sheet, which binds to the nucleic acids. The glass fiber sheet disperses throughout the liquid upon mixing, enhancing the binding interaction with nucleic acids.

We compared eDNA yields from samples processed in the presence or absence of BAC (0.001% w/v final) using three methods: QuickConc, glass fiber filter (Advantec GA‐55), and Sterivex (Millipore SVHV010RS). Based on our investigation into the optimal BAC concentration from the perspectives of eDNA yield and operational feasibility, comparisons were only conducted with a BAC concentration of 0.001% (Figure S1). The extraction conditions for the glass fiber filter and QuickConc were determined after a preliminary investigation of various extraction buffer compositions (Figure S2). The detailed protocol is described below:

- *QuickConc*: The concentration process for QuickConc is illustrated in Figure 2; it was commercialized as a result of the present study. Briefly, one piece of glass fiber sheet (1 cm square) was added to a 2‐L bag filled with environmental water. The bag was stirred vigorously and then allowed to rest for 1 min. During this process, the glass fiber sheet disintegrated into individual fibers, dispersing throughout the water. These dispersed fibers were then collected using a mesh filter and transferred to a 2 mL tube. We added 400 μL of ATL buffer and 40 μL of proteinase K and then incubated it at 56°C for 30 min. Following the incubation, 800 μL of 100% ethanol was added and mixed before purification via a spin column. The DNA was purified using the DNeasy Blood & Tissue Kit (Qiagen).
- *Glass fiber filter*: Water samples were filtered under vacuum through a 0.6 μm nominal pore size glass fiber filter (Advantec GA‐55). The water filtration and eDNA extraction processes followed a slight modification of the process outlined in the environmental DNA sampling and experiment manual published by The eDNA Society (2019). The filter was placed into a Salivette tube, and 400 μL of ATL buffer and 40 μL of proteinase K were added and then incubated at 56°C for 30 min, followed by centrifugation at 3000 *g* for 3 min. After adding 220 μL of nuclease‐free water and another centrifugation, 400 μL of 100% ethanol was mixed with the solution before purification via a spin column, according to the DNeasy Blood & Tissue Kit (Qiagen) protocol.
- *Sterivex*: We used the Sterivex cartridge extraction method, as described previously (Wu and Minamoto 2023). Briefly, 1000 μL of ATL buffer, 495 μL of PBS, 455 μL of AL buffer, and 50 μL of proteinase were added to Sterivex with filtered samples, followed by a 30‐min incubation at 56°C. The extracted DNA was then collected using a 2 mL syringe, 1000 μL of ethanol was added, and it was purified using a spin column.

**FIGURE 3:**
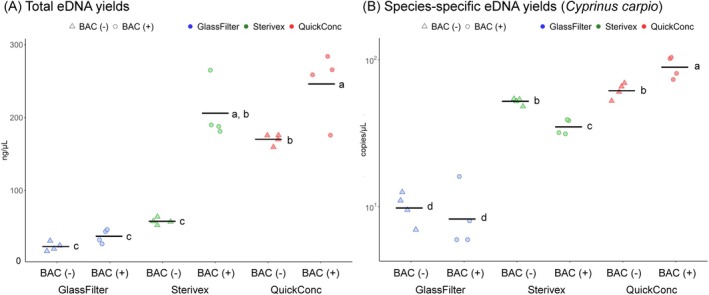
Comparison of the eDNA yields with and without benzalkonium chloride (BAC) using different concentration methods. The experiment was conducted using 400 mL of river water concentrated using QuickConc, compared to the glass filter and Sterivex methods. For BAC (+), BAC was added to the environmental water to a final concentration of 0.001%. Panel (A) shows the total eDNA yields in extracted DNA, and Panel (B) shows the results for *Cyprinus carpio* eDNA in 400 mL of river water using a log scale for the *y*‐axis. Each dot represents a sample; the midlines indicate the averages. Statistical analyses of the differences among groups were performed using Tukey's honest significant difference (HSD) test. The different letters are significantly different factor levels (*p* < 0.05) according to Tukey's HSD.

All extracts were eluted in 100 μL and measured by Nanodrop‐1000 (Thermo Fisher Scientific). We then performed species‐specific qPCR for *Cyprinus carpio* and statistical analyses following the protocols described below (see 2.3 and 2.5 for details). The results are summarized in Figure 3.

### 2.2 Experiment 2: Comparison of QuickConc, a Glass Fiber Filter, and Sterivex in Three Environments

#### 2.2.1 Sample Collection for Experiment 2

Water sampling was conducted across three aquatic environments in Japan, including river surface water from the Muko River (34°45′32.1″N 135°22′26.8″E), collected on April 1, 2024; sea surface water from the Kobe Port (34°38′27.9″N, 135°13′35.8″E), collected on March 28, 2024; and pond water (34°44′31.6″N 135°05′48.5″E), collected on April 11, 2024. Figure 1 demonstrates the experiment's design. Sample volumes of 400 mL, 1 L, and 70 mL per sample were collected from the river, sea, and pond, respectively. Four replicates plus one field blank were taken per site. Each sample was immediately processed using QuickConc, a glass fiber filter, or Sterivex after the addition of 0.001% w/v BAC (see right‐hand diagram in Figure 1).

#### 2.2.2 Concentration and DNA Extraction for Experiment 2

We used the same procedures as in Experiment 1, with the modification that all methods included the addition of BAC to a final concentration of 0.001%, based on the results shown in Figure 3. Each extract was eluted in a final volume of 100 μL and stored at −80°C until further analysis. Total eDNA concentration was quantified via Nanodrop‐1000 (Thermo Fisher Scientific).

### 2.3 qPCR Analysis

For comparisons among concentration methods, species‐specific qPCR assays were performed to quantify the eDNA of *Acanthopagrus schlegelii* , *C. carpio* , and *Misgurnus anguillicaudatus* in the river, sea, and pond water samples, respectively. These species were selected because previous surveys confirmed their presence in sufficient quantities in each environment. Primers and TaqMan probe designs were aligned with previous studies (Jo et al. 2020; Takahara et al. 2012; Takahashi et al. 2020), ensuring specificity and reliability (Table S1). Each qPCR reaction comprised a 20 μL final volume, including 900 nM primers, 125 nM TaqMan probe, 1× Environmental Master Mix 2.0, and 0.1 μL AmpErase Uracil N‐Glycosylase (Thermo Fisher Scientific), with 2 μL of the eDNA extract. Thermal cycling was initiated with a 2‐min step at 50°C and a 10‐min polymerase activation at 95°C, followed by 55 cycles of 15 s at 95°C and 1 min at 60°C. Calibration curves were established using a 10‐fold serial dilution series of standards (five levels of concentrations: 10–100,000 copies per reaction). Samples from the different concentration methods were analyzed on the same qPCR plate along with the corresponding standard curve. All qPCR experiments, including standards, eDNA samples, filtration blanks, and PCR‐negative controls, were performed in triplicate wells within a single qPCR plate, ensuring the accuracy of DNA concentration calculations for the environmental samples.

### 2.4 Amplicon Library Preparation, MiSeq Sequencing, and Data Processing

To evaluate the effect of different concentration methods on the number of detected species and community composition, metabarcoding analyses were conducted for fish eDNA. Our sampling sites included the river and the sea, but notably excluded the pond samples from the sequencing process. The pond samples were excluded because this pond, formerly a rice paddy in an isolated area, did not represent broad aquatic ecosystems and only two fish species are known to be present. After DNA extraction, subsequent procedures of amplicon library preparation and NGS were performed by the Bioengineering Lab. Co. Ltd., in Kanagawa, Japan. The DNeasy PowerClean Pro Cleanup Kit (Qiagen) was used to remove PCR‐inhibitory substances, and the elution of DNA was performed in a final volume of 50 μL. The library construction began with a two‐step tailed PCR method using four primer sets (Table S3), which were partially modified fish universal PCR primers, MiFish, developed by Miya et al. (2015) to amplify eDNA from fish species. The first‐round PCR reaction was conducted in a 10 μL mixture containing 0.375 U ExTaq HS (TaKaRa), 1× ExTaq Buffer, 0.2 mM of each dNTP, 0.5 μM of each primer, 1 μL of the eDNA template, and ultrapure water. The thermal cycle profile was 94°C for 2 min, followed by 35 cycles of 20 s at 94°C, 15 s at 65°C, and 20 s at 72°C, with a final extension of 5 min at 72°C. After purification of the first PCR products using VAHTS DNA Clean Beads (Vazyme), the second‐round PCR had a 10 μL total reaction volume, including 0.2 U KOD FX Neo (TOYOBO), 1 × PCR Buffer, 0.4 mM of each dNTP, 0.25 μM each primer, 2 ng of the first PCR product, and ultrapure water. The second PCR's thermal cycles profile consisted of 94°C for 2 min, 12 cycles at 98°C for 10 s, 60°C for 30 s, and 68°C for 30 s, with a final extension at 68°C for 2 min. PCR products were purified using VAHTS DNA Clean Beads (Vazyme), and then the paired‐end sequence libraries were completed. The quality of the constructed library was verified using a fragment analyzer and the dsDNA 915 Reagent Kit (Agilent Technologies), after which sequencing was conducted on the MiSeq system using the MiSeq Reagent Kit v3 (Illumina) under conditions of 2 × 300 bp. Only read sequences, which were fully matched with primer sequences at the beginning, were extracted using the fastq barcode splitter in the FASTX Toolkit (ver. 0.0.14). FASTX‐Toolkit's fastx_trimmer was used to remove the primer sequences and the last 120 bases at the 3′ end from the extracted reads. Subsequently, sequences with a quality score below 20 were filtered out using Sickle (ver. 1.33), and any sequences shorter than 40 bases, along with their paired reads, were discarded. Following this, the DADA2 plugin in Qiime2 (ver. 2024.2) was used to remove chimeric and noisy sequences, after which representative sequences and an ASV table were generated. For MiFish analyses, the obtained ASV table was analyzed with USEARCH v 10.0.240 (Edgar 2010), according to the method demonstrated by Wu et al. (2021). The mitochondrial genome database MitoFish (ver. 3.90) was used to assign taxonomic names to ASVs. The raw sequence data were uploaded to Dryad (see Data Availability Statement).

### 2.5 Statistical Analysis

Each analysis was performed using R ver. 4.3.3 (R Development Core Team 2024), with the vegan package version 2.5‐6 (Oksanen et al. 2019) and the lme4 package version 1.1‐21 (Bates et al. 2015). To compare the total nucleic acid yield or species‐specific DNA concentrations in environmental waters across different filtration methods, statistical analyses were conducted using one‐way ANOVA, followed by Tukey's honest significant difference (HSD) post hoc test for pairwise comparisons. For metabarcoding data, we conducted three analyses: comparison of the number of species, evaluation of within‐method β diversity, and non‐metric multidimensional scaling (NMDS). To estimate whether concentration methods influenced the number of fish species detected by metabarcoding, read data were converted to presence/absence, and a generalized linear mixed model with a Poisson distribution was used. When the effect of concentration methods was significant, we employed Tukey's multiple comparison test. To assess how concentration methods affect estimates of β diversity, we calculated within‐method β diversity, hereafter termed “pseudo β diversity,” representing β diversity between independent replicates of the same sample. Pseudo β diversity was calculated by dividing the total number of species detected (γ) by the number of species detected within each sample (α) for each method. The following analyses were performed individually for un‐rarefied and rarefied data, which were obtained by unifying the number of total reads to the fewest reads through rarefying, using the vegan R package. To visualize the dissimilarities in fish composition due to presence/absence, NMDS was performed using the Jaccard method with 10,000 permutations. In this model, absence was assigned a value of 0, and presence was assigned a value of 1. Additionally, to compare fish composition among the three concentration methods, we performed permutational multivariate analysis of variance (PERMANOVA) and multivariate dispersion (PERMDISP) with 10,000 permutations using the “adonis” and “betadisper” functions of the vegan package, respectively. We used ggplot2 package version 3.1.1 to construct some of the graphs.

## 3 Results

### 3.1 Experiment 1: The Impact of BAC on eDNA Yield (River Water)

To demonstrate that BAC enhances DNA yield when used with dispersed glass fibers, we analyzed total eDNA yields and *C. carpio* eDNA copy numbers for QuickConc, a glass fiber filter, and Sterivex, with or without BAC. As shown in Figure 3 and Table S4, QuickConc with BAC showed roughly 1.2–6.6 times more total eDNA than the glass fiber filtration and Sterivex methods (one‐way ANOVA with Tukey's HSD post hoc test, *p* < 0.001; Figure 3A). Species‐specific qPCR results showed that QuickConc with BAC detected 1.7–8.8 times higher copy numbers compared to the other methods (one‐way ANOVA with Tukey's HSD post hoc test, *p* < 0.001; Figure 3B). Given these results, we employed BAC (0.001%) in QuickConc for the subsequent experiment.

### 3.2 Experiment 2: A Comparison Among QuickConc, Glass Fiber Filters, and Sterivex

**FIGURE 4:**
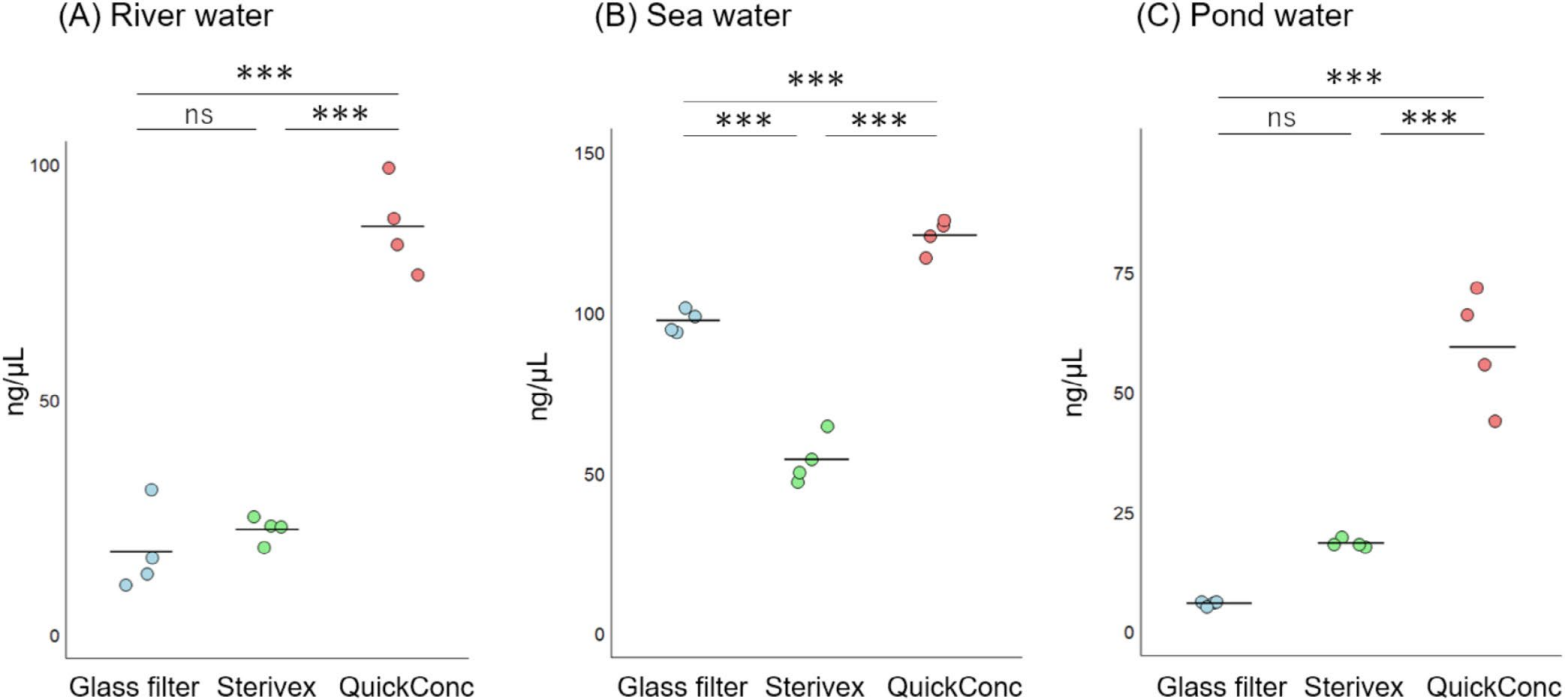
Comparisons of the DNA yields using different concentration methods. Panels (A–C), respectively, show the total eDNA yields in extracted DNA from 400 mL of river water, 1 L of seawater, and 70 mL of pond water. For all types of environmental waters, the QuickConc method consistently resulted in significantly higher concentrations of eDNA compared to the glass filter and Sterivex methods. Each dot represents a sample; the midlines indicate the averages. Statistical analyses of the differences among groups were performed using Tukey's honest significant difference test (^ns^ *p* ≧ 0.05, ***p < 0.001).

**FIGURE 5:**
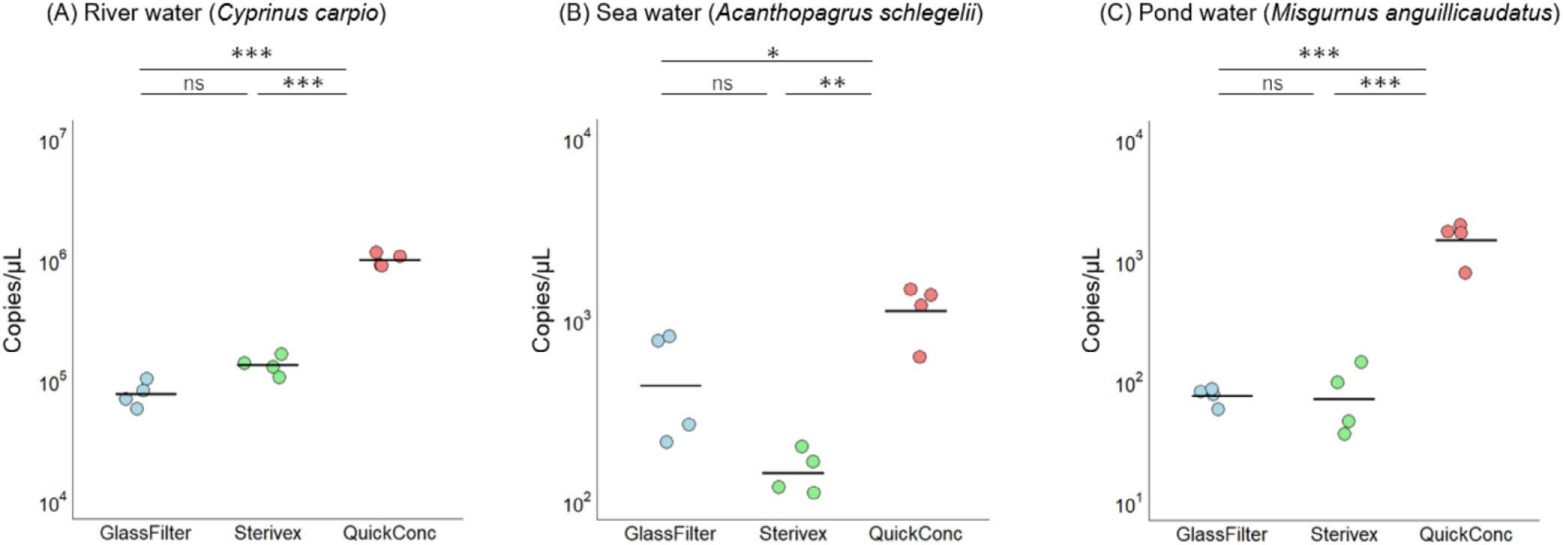
Quantitative comparison of DNA concentrations in extracted DNA for each target species using different concentration methods. Panels (A–C) show the results for *Cyprinus carpio* eDNA in 400 mL of river water, *Acanthopagrus schlegelii* eDNA in 1 L of seawater, and *Misgurnus anguillicaudatus* eDNA in 70 mL of pond water, respectively. Each dot represents a sample; the midlines indicate the averages using a log scale for the *y*‐axis. For all types of environmental waters, the concentration of DNA from the target fish species was significantly higher when using the QuickConc method, compared to the glass filter and Sterivex methods (Tukey's HSD; ^ns^ *p* ≧ 0.05, *p < 0.05, **p < 0.01, and ***p < 0.001).

Next, we evaluated the performance of nucleic acid yields enhanced by BAC, in comparison to other methods using glass fiber filters and Sterivex (Figure 4). The QuickConc method yielded significantly more total eDNA than the other methods in all aquatic environments. Specifically, in the river water samples, it recovered about three times as much total eDNA as the glass filter and Sterivex methods (ANOVA, *p* < 0.001 and Tukey's HSD, *p* < 0.001, Figure 4A). In the sea water samples, QuickConc yielded roughly 1.3–2.3 times more total eDNA (ANOVA, *p* < 0.001 and Tukey's HSD, *p* < 0.001, Figure 4B), and in the pond water samples, it recovered about three times more total eDNA (ANOVA, *p* < 0.001 and Tukey's HSD, *p* < 0.001, Figure 4C). Subsequently, qPCR analyses of specific species were compared with these methods. The overall PCR efficiencies, *R* ^2^ values of the standard curves, and copy numbers are shown in Tables S2 and S4. Specifically, the *R* ^2^ values were all above 0.99, and the amplification efficiencies were 110%, 101%, and 91% for *A. schlegelii* , *C. carpio* , and *M. anguillicaudatus* , respectively. All qPCR‐negative controls and field blanks showed no amplification, confirming the absence of contamination. The copy numbers for *A. schlegelii* , *C. carpio* , and *M. anguillicaudatus* were significantly higher when using the QuickConc method compared to other methods. Specifically, in the river water, the copy number for *C. carpio* using QuickConc was approximately 10 times higher than with Sterivex (ANOVA, *p* < 0.001 and Tukey's HSD, *p* < 0.001, Figure 5A). For *A. schlegelii* in sea water, QuickConc yielded about 8 times higher copy numbers than Sterivex (*p* < 0.05, Figure 5B) and about 2.3 times higher than the glass filter method (*p* < 0.01, Figure 5B). Similarly, for *M. anguillicaudatus* in pond water, the QuickConc method resulted in a copy number more than 10 times higher than with Sterivex and the glass fiber filters (*p* < 0.001, Figure 5C).

**FIGURE 6:**
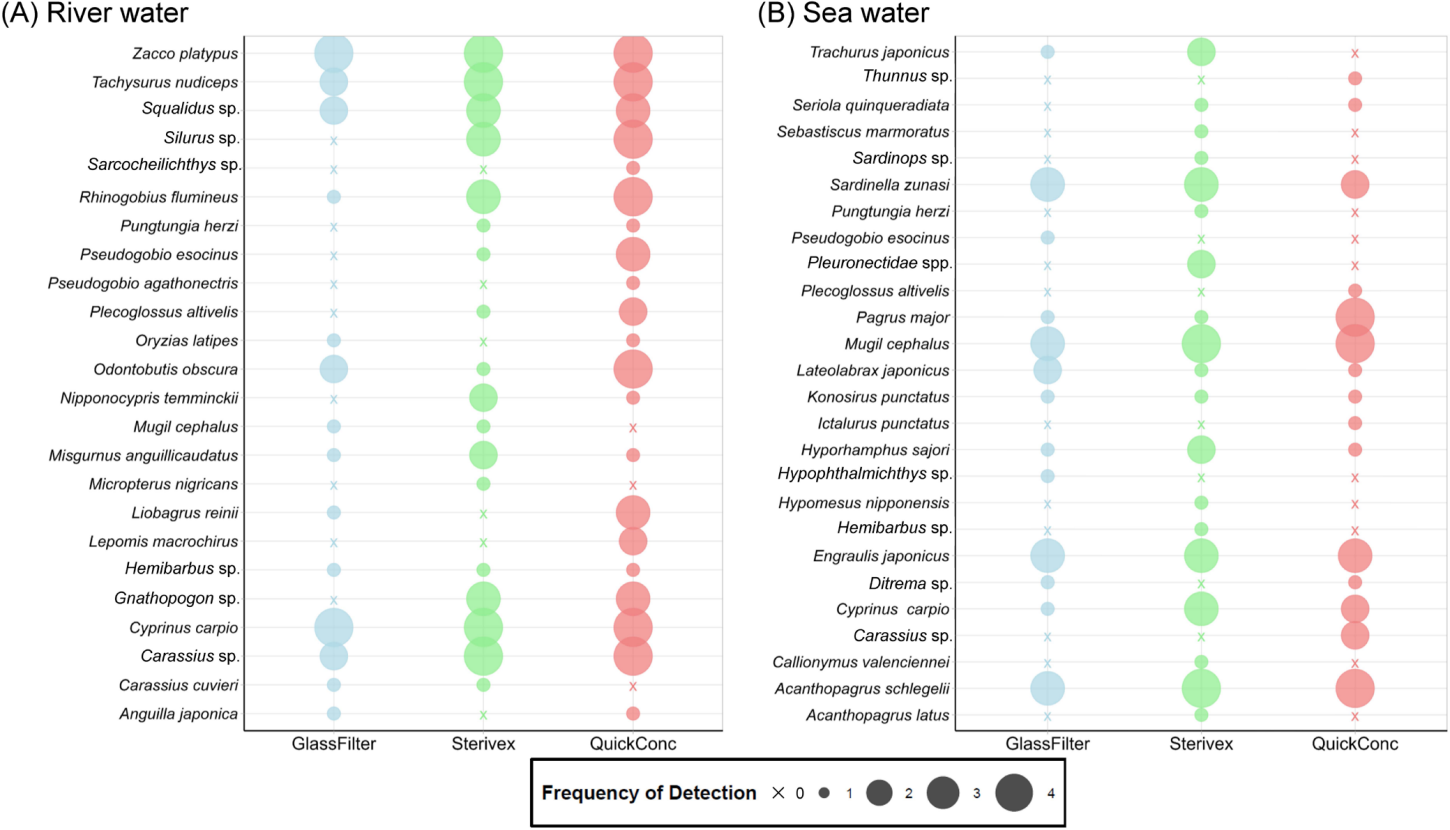
Comparison of the number of fish detected using different concentration methods. The bubble chart shows the quantitative comparison of fish species detected across three concentration methods: Glass filter, Sterivex, and QuickConc. These results are based on eDNA extracted from 400 mL of river water and 1 L of seawater and categorized by the detection frequency within these environmental samples. The vertical axis lists the fish species identified, while the circle size indicates the frequency of species detected in replicate sampling. The absence of detection is noted with an “x.”

**FIGURE 7:**
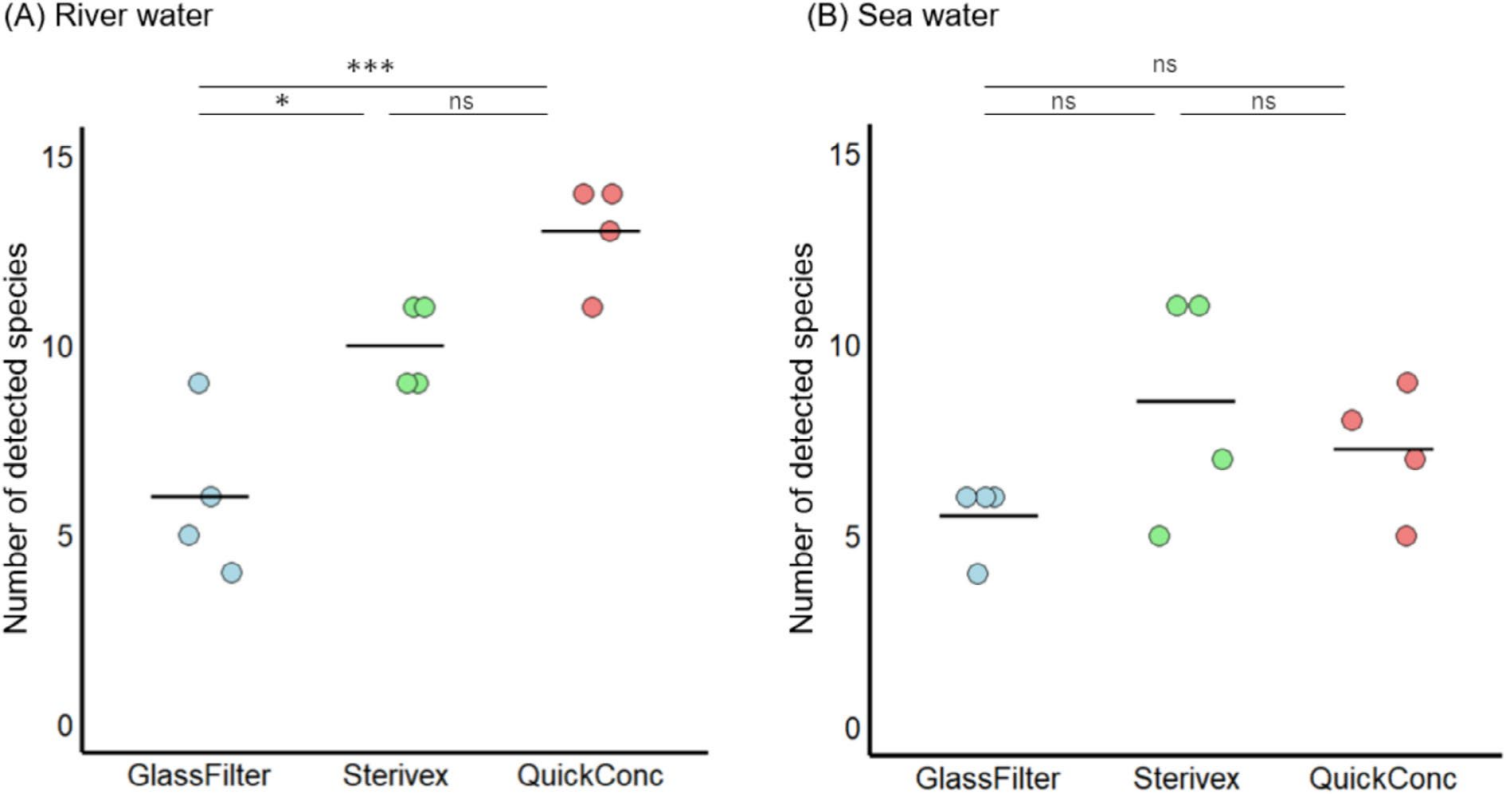
The total number and composition of fish species detected using different methods. Each dot represents a sample; the midlines indicate the averages. Statistical analyses of the differences among groups were performed using Tukey's honest significant difference test (^ns^ *p* ≧ 0.05, *p < 0.05, ***p < 0.001).

**FIGURE 8:**
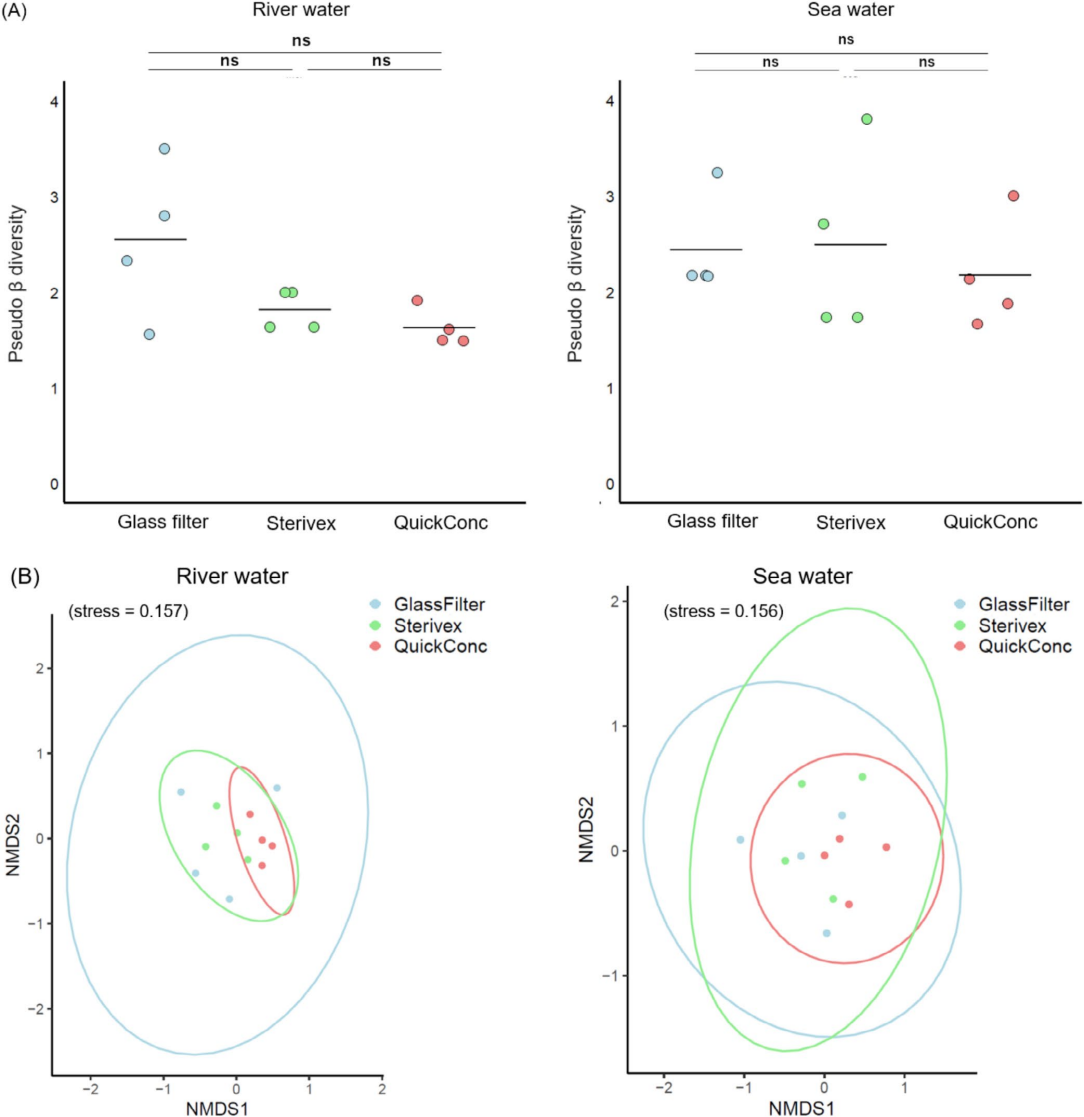
Pseudo β diversity and composition of fish species detected using different preservation methods. Panel (A) shows pseudo β diversity, which we define as dissimilarity between replicates of the same sample. Each dot represents a sample; the midlines indicate the averages. Statistical analyses of the differences among groups were performed using Tukey's HSD test (^ns^ *p* ≧ 0.05). Panel (B) shows a non‐metric multidimensional scaling analysis with rarefaction, which was conducted to assess the dissimilarity in estimated fish communities' compositions, obtained via different concentration methods. The circle represents confidence intervals around the central tendency of the data points for each method: Blue for glass filter, green for Sterivex, and red for QuickConc.

Finally, we performed metabarcoding analyses using MiFish primers. A total of 1,781,738 and 3,272,945 reads were obtained for the river and sea waters, respectively, which were reduced to 853,367 and 981,290 after filtering (Table S5, Figure S3). Fish DNA was not detected from the field blank or PCR‐negative controls. In the river samples, totals of 14, 18, and 21 fish species were detected with glass fiber filters, Sterivex, and QuickConc, respectively (Figure 6). Similarly, in the sea water samples, totals of 13, 19, and 15 fish species were detected, respectively. The numbers of fish species detected in river water were significantly higher with QuickConc compared to the glass fiber filters, but there was no significant difference observed when compared to Sterivex (Tukey's HSD, *p* < 0.001 and *p* = 0.194, Figure 7A). By contrast, the number of fish species detected from sea water did not differ significantly among the concentration methods (Tukey's HSD, *p* = 0.498 and *p* = 0.731, Figure 7B). To evaluate how these concentration methods affect estimates of sample β diversity, we calculated pseudo β diversity within each method. Although there was a decreasing trend for the QuickConc method, there were no significant differences among any of the methods in the river and sea waters (Figure 8A). Next, to assess differences in community composition among methods, we performed NMDS analyses with rarefaction and statistical analyses including PERMANOVA and PERMDISP. These results demonstrated that significant differences in community composition were observed in the river water samples (PERMANOVA, *p* = 0.026; PERMDISP, *p* = 0.205, Figure 8B), while no significant differences in community composition were observed in the seawater samples (PERMANOVA, *p* = 0.638; PERMDISP, *p* = 0.655, Figure 8B). By contrast, when using non‐rarefied data from both river and sea water samples, the trends in community composition remained consistent with the rarefied data (Figure S4).

## 4 Discussion

This study introduced QuickConc, a novel eDNA capture method designed for rapid and sensitive analysis. We compared QuickConc to the traditional glass fiber filtration and Sterivex methods, quantifying both total eDNA and species‐specific qPCR targets in river, sea, and pond water samples. QuickConc consistently yielded high eDNA concentrations (Figures 3, 4, 5), suggesting it is a promising option for biodiversity monitoring and conservation applications. While absolute eDNA quantities varied between Experiments 1 and 2 due to differences in river water and sampling dates, the relative trends in eDNA extraction efficacy remained consistent across both experiments, with QuickConc consistently yielding high amounts of total eDNA and species‐specific eDNA compared to the commonly employed protocols under our experimental conditions.

NMDS analyses showed a trend in which QuickConc results clustered within the variability observed for glass fiber filter and Sterivex methods. This, along with a trend of lower pseudo β‐diversity, suggests that QuickConc may exhibit less variability in detected species, potentially contributing to reproducible eDNA quantification. Future studies with larger sample sizes, especially in high‐diversity or low‐biomass environments, are needed to further validate QuickConc's performance.

We propose three primary mechanisms to explain QuickConc's improved eDNA capture efficiency. First, BAC likely acts as a DNA‐binding enhancer. The established mechanism for DNA binding to silica surfaces involves two primary interactions: the phosphate groups of DNA bind to the silanol groups on the silica surface, and the hydrophobic regions of DNA interact with the hydrophobic domains of the silica (Shi et al. 2015; Wu et al. 2018). Furthermore, positively charged molecules are known to function as binding agents (Bag et al. 2021). Based on this, we propose that BAC, with its positive charge and side chains, enhances DNA capture by forming molecular bridges between silica and DNA, thus facilitating both electrostatic binding and hydrophobic interactions. Second, BAC's surfactant properties may promote DNA release from cells and organelles. Since much eDNA is protected within cellular structures (Power et al. 2023; Thamke et al. 2024), this release could significantly improve capture efficiency. Third, BAC has the effect of dissociating DNA from the surface of suspended particles (Huang et al. 2023; Husale et al. 2008; Thamke et al. 2024). Importantly, the observed increase in sensitivity with BAC (Figure 3) is not understood to be due to a preservative effect (Takahara et al. 2020), as all eDNA extractions were conducted immediately after sampling. Therefore, BAC likely increases the total dissolved eDNA by dissociating it from particles and intracellular compartments.

We observed the influence of BAC on species‐specific eDNA capture and extraction efficiency specifically when using dispersed glass fibers, but not glass fiber filters or Sterivex (Figure 3B). This difference likely stems from the distinct physical properties of the methods. Dispersed glass fibers are mobile in solution, providing greater flexibility for BAC‐mediated DNA binding. In contrast, traditional filters have limited passage time, while finely dispersed glass fibers possess a higher surface area, providing more opportunities for interaction with nucleic acids. These factors likely explain the enhanced total eDNA recovery observed only with QuickConc. Conversely, Sterivex, employing a poly vinylidene di‐fluoride membrane relying on physical filtration with their limited interaction time with DNA, did not experience significant BAC‐mediated improvements in species‐specific DNA yield because BAC does not enhance the physical filtration of eDNA through the Sterivex membrane. In Sterivex samples, while the addition of BAC showed no change in species‐specific eDNA quantities, the total eDNA yields were increased. This might also be influenced by the membrane material used in Sterivex filters, as well as the specific characteristics of the environmental sample itself. The observed variation in the relative eDNA yields between Experiments 1 and 2 in river water, and among different environmental water types (river, sea, and pond) in Experiment 2, strongly suggests that the efficiency of eDNA concentration methods can be significantly influenced by the environmental sample. Factors such as water turbidity, salinity, and the presence of inhibitory substances may interact differently with the membrane materials and extraction protocols of each method, leading to differential recovery rates of eDNA. However, the exact mechanism and the impact of environmental water differences remain unclear and warrant further investigation in future studies. Additionally, spike‐in recovery experiments confirmed QuickConc's consistently higher eDNA recovery rates across all stages of concentration and extraction (Figure S5A). In the spike‐in test during the capture step, the recovery rate of QuickConc was approximately 15% higher compared to other methods (Figure S5B,C). Notably, QuickConc showed higher recovery than other methods when the standard DNA was spiked before elution with ProK treatment. This suggests QuickConc shows high eDNA elution efficiency under our experimental conditions. We attribute this to the QuickConc method, which allows the entire glass sheet, with its bound eDNA, to be transferred directly to the silica column, facilitating more complete nucleic acid transfer during elution. These findings highlight the importance of silica mobility and accessibility for maximizing BAC's DNA capture and extraction enhancing capabilities, particularly in applications employing suspended glass fiber filters.

Unlike traditional filters (pore sizes: 0.7–3.0 μm for glass fiber, 0.22–0.45 μm for Sterivex), QuickConc uses a mesh filter with a larger pore size (20–175 μm) to capture the dispersed glass fiber sheets. This enables QuickConc to achieve high‐speed filtration without the need for high pressure. The bag‐type concentration system also allows for shaking to alleviate clogging, resulting in more efficient and faster concentration with reduced clogging. We successfully applied QuickConc to highly turbid pond water. As shown in Figures 3 and 4, we tested 70 mL of pond water, which is the maximum volume that can be manually passed through Sterivex, leading us to concentrate eDNA from the same volume across all methods for consistency. However, QuickConc was able to concentrate at least 500 mL of pond water, indicating that the method can handle larger volumes without clogging and may result in greater sensitivity. When analyzing data at different volumes, it is important to interpret the results by adjusting for volume. Furthermore, QuickConc was effective for both clear and high‐turbidity samples tested in this study, suggesting its potential utility across a range of water conditions. However, while QuickConc's mesh filter mitigates clogging and allows for greater filtration volumes, it does not entirely resolve the issue in highly turbid samples. For extremely turbid environmental water where filtration remains challenging, it may be necessary to consider alternative approaches such as directly extracting eDNA from soil or sediment instead of the water sample.

While QuickConc consistently yielded high total eDNA concentrations, the impact on metabarcoding results varied across environments. In river water, the increased total eDNA concentration with QuickConc resulted in significantly different fish community profiles, compared to the other methods (Figures 7A and 8A), highlighting its ability to capture a broader range of species present in the environment. However, in marine samples, the number of detected fish species and community composition did not differ significantly among the methods (Figures 7B and 8B), despite the higher total eDNA yield with QuickConc. Several factors may explain the lack of significant differences in marine samples. First, the relatively low level of fish diversity at the marine sampling site may have resulted in no differences in detectable species, regardless of the concentration method. Second, different capture mechanisms could influence the types of eDNA recovered. While Sterivex and glass fiber filters rely on size‐based filtration, potentially capturing eDNA alongside debris, QuickConc likely employs charge‐based interactions for eDNA capture. This difference is supported by the observed variations in community composition in the river samples (Figure 8), suggesting that the capture mechanism can influence the detected species profile.

Furthermore, among the fish species listed in the Red List set issued by the Japanese Ministry of the Environment (https://www.env.go.jp/content/900515981.pdf) (in Japanese), *Anguilla japonica, Carassius cuvieri, Liobagrus reinii, Misgurnus anguillicaudatus*, and *Oryzias latipes* were detected (Figure 6). Particularly in the case of *Liobagrus reinii* , while detection rates were 1 out of 4 and 0 out of 4, with glass fiber filter and Sterivex, respectively, the QuickConc method detected it in 3 out of 4 samples. Even for rare fish species with low population numbers, using the QuickConc method to enhance the total eDNA concentration efficiency can be expected to enable a more stable detection of specific fish species.

Using BAC in environmental applications requires a risk assessment. Historically, BAC has been widely used in disinfectants, and its risk has been extensively evaluated due to its potential detrimental effects on both environmental and human health (Merchel Piovesan Pereira and Tagkopoulos 2019). According to the Globally Harmonized System classification, BAC poses concerns in terms of aquatic toxicity. Importantly, the BAC concentration used in QuickConc (0.001%) is significantly lower than in disinfectants (0.01%–1%) (Artasensi et al. 2021). Our OECD‐compliant risk assessment indicates a hazard quotient below 1, suggesting minimal environmental risk under typical conditions. However, risk assessment results can vary depending on various environmental factors, such as water body type, flow rate, and local ecosystem characteristics. Moreover, the discharge of BAC might be subject to varying regulations across different jurisdictions. With the above in mind, it is recommended that practitioners either discharge the treated water containing low BAC concentrations to the sewage system under controlled conditions or transport it back to a laboratory setting for appropriate treatment to mitigate any potential environmental impact.

QuickConc is proposed to increase eDNA yields by dissociating eDNA bound to suspended particles or intracellular eDNA into the water with the effect of BAC. However, this process may also affect inhibitory compounds in environmental water, potentially leading to PCR inhibition. In our settings, QuickConc consistently demonstrated the highest total‐ and targeted‐eDNA yield (Figure 4). In the river water samples, the high yield of the total eDNA resulted in the increased number of species detected and significant differences in community compositions (Figures 7A and 8A). These findings suggest that samples captured with QuickConc are able to recover more total eDNA, thereby enabling the detection of trace amounts of specific eDNA. However, it is important to note that PCR inhibition may occur more strongly in certain samples. Further detailed research is needed to address the above uncertainties.

While the results of this study are promising, it is important to acknowledge its limitations. First, our experiments were conducted at specific sites in Japan (river, sea, and pond), and the performance of QuickConc could vary in other aquatic environments with different physicochemical properties, such as varying pH, organic matter content, or the presence of different types of PCR inhibitors. Second, our protocol utilizes Qiagen's Buffer ATL. Although it was effective for eDNA recovery in our tests, it should be noted that the use of different extraction buffer systems across the three methods (QuickConc, glass fiber filter, and Sterivex) may have resulted in differences in extraction efficiency. Therefore, while QuickConc demonstrated high eDNA yield and detection sensitivity in our settings, further validation across a broader range of environmental conditions and comparative methods is necessary to fully understand its performance and applicability.

QuickConc is designed to be user‐friendly and accessible to a broad range of individuals, including those without extensive scientific training. This is particularly relevant for low‐resource settings, such as those found in low‐ and middle‐income countries. The concentration procedure requires no specialized technique or power, making it feasible for users of all ages and skill levels to participate in eDNA analysis. This design facilitates citizen science initiatives, enabling a wider range of people to contribute to environmental monitoring and research.

## Author Contributions

**Tomohiro Kuroita:** conceptualization (equal), data curation (equal), formal analysis (equal), investigation (equal), methodology (equal), visualization (equal), writing – original draft (lead), writing – review and editing (equal). **Qianqian Wu:** investigation (equal), writing – review and editing (equal). **Ryo Iwamoto:** conceptualization (equal), methodology (equal), writing – review and editing (equal). **Toshifumi Minamoto:** conceptualization (equal), funding acquisition (equal), resources (equal), supervision (equal), writing – review and editing (equal).

## Conflicts of Interest

The authors declare the following financial interests/personal relationships which may be considered as potential competing interests: T.K. and R.I. report employment relationships with Shionogi and Co Ltd. and are the inventors of patents for the QuickConc. T.M. is an inventor of the patent for the use of benzalkonium chloride for eDNA preservation.

## Supporting information


**Table S1:** Nucleotide sequences of primers and probes.
**Table S2:** R2 values, slopes, and Y intercepts of the calibration curves, and the PCR efficiencies for each target species.
**Table S3:** Nucleotide sequences of metabarcoding primer.
**Table S4:** Quantitative real‐time PCR (qPCR) for each target species with different preservation methods. Environmental DNA (eDNA) concentration was assessed using qPCR.
**Table S5:** Detailed list of fish species detected from different concentration methods by eDNA metabarcoding.
**Figure S1:** Evaluation of BAC concentration. The experiment was conducted using 400 mL of river water concentrated using QuickConc, compared to the concentration of BAC. BAC was added to the environmental water to a final concentration of 0%, 0.0001%, 0.001% and 0.01%. For samples with a BAC concentration of 0.01%, excessive foaming occurred during the eDNA concentration process, making it impossible to collect all glass sheets. Therefore, subsequent analyses were conducted using only the recovered glass sheets. Panel (A) shows the total eDNA yields in extracted DNA, and Panel (B) shows the results for Cyprinus carpio eDNA recovered from 400 mL of river water using log‐scale for the y‐axis. Each dot represents a sample; the midlines indicate the averages. Statistical analyses of the differences among groups were performed using Dunnett's test (nsp ≧ 0.05, ***p < 0.001).
**Figure S2:** Comparison of the eDNA yields using different extraction buffer. The experiment was conducted using 400 mL of river water concentrated using QuickConc and the glass filter methods. For each method, total eDNA and species‐specific eDNA yields were compared under three extraction conditions: using only AL, only ATL, or both AL and ATL for extraction. When both AL and ATL were used, 400 μL of ATL was used during the ProK treatment, and 400 μL of AL was added before ethanol precipitation. Panel (A) shows the total eDNA yields in extracted DNA, and Panel (B) shows the results for *Cyprinus carpio* eDNA recovered from 400 mL of river water using log‐scale for the *y*‐axis. Each dot represents a sample; the midlines indicate the averages. Statistical analyses of the differences among groups were performed using Tukey's honest significant difference (HSD) test. The different letters are significantly different factor levels (^ns^ *p* ≧ 0.05, *p < 0.05, **p < 0.01, ***p < 0.001) according to Tukey's HSD.
**Figure S3:** eDNA metabarcoding species accumulation curves. Species accumulation curves for river (A) and sea (B) water show how many species on average are detected with increasing number of reads across three concentration methods: Glass filter (blue), Sterivex (green), and QuickConc (red).
**Figure S4:** Composition of fish species detected using different preservation methods. A non‐metric multidimensional scaling (NMDS) analysis with non‐rarefied data was conducted to assess the dissimilarity in estimated fish community composition obtained via different concentration methods. The circle represents confidence intervals around the central tendency of the data points for each method: blue for glass filter, green for Sterivex, and red for QuickConc.
**Figure S5:** (A). Experiment design diagram of spike‐in test. Volumes of 400 mL TBS buffer per sample were used, respectively. Four replicates per condition were conducted, and each was subjected to DNA extraction. Spike DNA consisted of chemically synthesized double‐stranded DNA containing sequences for Cyprinus carpio and Acanthopagrus schlegelii, as detailed in Table S1. The recovery rate of spike DNA was evaluated across three spike‐in groups: (1) before concentration, (2) before lysis by ProK, and (3) immediately before addition to the silica column. DNA was added to each group at a concentration of 105 copies per sample.
**Figure S5:** (B, C). Comparison of the DNA recovery rate. The experiment was conducted using 400 mL of TBS buffer concentrated using QuickConc, compared to the glass filter and Sterivex methods. Panel (B) shows the recovery rate for Cyprinus carpio DNA using log‐scale for the y‐axis, and Panel (C) shows the recovery rate for Acanthopagrus schlegelii DNA using log‐scale for the y‐axis. Each dot represents a sample; the midlines indicate the averages. Statistical analyses of the differences among groups were performed using Tukey's honest significant difference (HSD) test. The different letters are significantly different factor levels (nsp ≧ 0.05, *p < 0.05, **p < 0.01, ***p < 0.001) according to Tukey's HSD.

## Data Availability

The raw sequence data were uploaded to Dryad (https://doi.org/10.5061/dryad.ttdz08m73). The authors confirm that the data supporting the findings of this study are available within the article and its Supporting Information.
