# Supplementary material for: QuickConc: A Rapid, Efficient, and Power‐Free eDNA Concentration Method With Cationic‐Assisted Capture
Source: Ecol Evol. 2025 Oct 8;15(10):e72269. doi: 10.1002/ece3.72269 (PMC12508262; doi:10.1002/ece3.72269)
Supplement: Supplementary file 1 — Table S1: Nucleotide sequences of primers and probes. Table S2: R2 values, slopes, and Y intercepts of the calibration curves, and the PCR efficiencies for each target species. Table S3: Nucleotide sequences of metabarcoding primer. Table S4: Quantitative real‐time PCR (qPCR) for each target species with different preservation methods. Environmental DNA (eDNA) concentration was assessed using qPCR. Table S5: Detailed list of fish species detected from different concentration methods by eDNA metabarcoding. Figure S1: Evaluation of BAC concentration. The experiment was conducted using 400 mL of river water concentrated using QuickConc, compared to the concentration of BAC. BAC was added to the environmental water to a final concentration of 0%, 0.0001%, 0.001% and 0.01%. For samples with a BAC concentration of 0.01%, excessive foaming occurred during the eDNA concentration process, making it impossible to collect all glass sheets. Therefore, subsequent analyses were conducted using only the recovered glass sheets. Panel (A) shows the total eDNA yields in extracted DNA, and Panel (B) shows the results for Cyprinus carpio eDNA recovered from 400 mL of river water using log‐scale for the y‐axis. Each dot represents a sample; the midlines indicate the averages. Statistical analyses of the differences among groups were performed using Dunnett's test (nsp ≧ 0.05, ***p < 0.001). Figure S2: Comparison of the eDNA yields using different extraction buffer. The experiment was conducted using 400 mL of river water concentrated using QuickConc and the glass filter methods. For each method, total eDNA and species‐specific eDNA yields were compared under three extraction conditions: using only AL, only ATL, or both AL and ATL for extraction. When both AL and ATL were used, 400 μL of ATL was used during the ProK treatment, and 400 μL of AL was added before ethanol precipitation. Panel (A) shows the total eDNA yields in extracted DNA, and Panel (B) shows the results for Cyprinus carpi [file ECE3-15-e72269-s001.docx]

**Supplementary information**

Table S1. Nucleotide sequences of primers and probes

| Assay | Primer/Probe | Name | Sequence (5’-3’) | Reference |
| --- | --- | --- | --- | --- |
| *Acanthopagrus schlegelii* | Forward | Asc_Cytb_F | CTGTCTGCCGTCCCCTACA |  |
|  | Reverse | Asc_Cytb_R | TATGGCGGCTACGATAAAAGGA | (Takahashi et al., 2020) |
|  | Probe | Asc_Cytb_Pr | FAM-TCAGTTGACAACGCAACCCTAACCCG-TAMRA |  |
| *Cyprinus carpio* L. | Forward | CpCyB_496F | GGTGGGTTCTCAGTAGACAATGC |  |
|  | Reverse | CpCyB_573R | GGCGGCAATAACAAATGGTAGT | (Takahara et al., 2012) |
|  | Probe | CpCyB_550p probe | FAM-CACTAACACGATTCTTCGCATTCCACTTCC-TAMRA |  |
| *Misgurnus anguillicaudatus* | Forward | Man-F | GGGTGTCCTAGCCCTTCTGTT |  |
|  | Reverse | Man-R | GTATGTCGGCGACTAGGGTTCA | (Jo et al., 2020) |
|  | Probe | Man-P | VIC-TGCCAATTCTCCACACATC-NFQ- MGB |  |

FAM: 6-carboxyfluorescein; MGB: minor groove binder; NFQ: nonfluorescent quencher; VIC: asymmetric xanthene; TAMRA: Tetramethylrhodamine

Table S2. R^2^ values, slopes, and Y intercepts of the calibration curves, and the PCR efficiencies for each target species.

| **Experiment** | **Target species** | **R^2^ value** | **Slope** | **Y-Intercepts** | **PCR efficiency%** |
| --- | --- | --- | --- | --- | --- |
| 1 | *Cyprinus carpio L.* | 0.999 | -3.386 | 39.2 | 97.4% |
| 2 | *Acanthopagrus schlegelii* | 0.999 | -3.099 | 38.9 | 110.2% |
|  | *Cyprinus carpio L.* | 0.997 | -3.296 | 40.1 | 101.1% |
|  | *Misgurnus anguillicaudatus* | 0.992 | -3.563 | 42.7 | 90.8% |

Table S3. Nucleotide sequences of metabarcoding primer

| PCR | Primer | name | Sequence (5-3’) | Reference |
| --- | --- | --- | --- | --- |
| 1st^1^ | Forward | MiFish-U-F | ACACTCTTTCCCTACACGACGCTCTTCCGATCTNNNNNNRGTTGGTAAATCTCGTGCCAGC | (Miya et al., 2015) |
|  | Reverse | MiFish-U-R | GTGACTGGAGTTCAGACGTGTGCTCTTCCGATCTNNNNNNGCATAGTGGGGTATCTAATCCTAGTTTG |  |
|  | Forward | MiFish-E-F | ACACTCTTTCCCTACACGACGCTCTTCCGATCTNNNNNNGTCGGTAAAACTCGTGCCAGC | (Miya et al., 2015) |
|  | Reverse | MiFish-E-R | GTGACTGGAGTTCAGACGTGTGCTCTTCCGATCTNNNNNNCATAGTGGGGTATCTAATCCCAGTTTG |  |
|  | Forward | MiFish-U2-F | ACACTCTTTCCCTACACGACGCTCTTCCGATCTNNNNNNGCCGGTAAAACTCGTGCCAGC | (The eDNA Society, 2019) |
|  | Reverse | MiFish-U2-R | GTGACTGGAGTTCAGACGTGTGCTCTTCCGATCTNNNNNNCATAGGAGGGTGTCTAATCCCCGTTTG |  |
|  | Forward | MiFish-Ayu&Wakasagi | ACACTCTTTCCCTACACGACGCTCTTCCGATCTGCCGGTTAATCTCGTGCCAGC | (Bioengineering Lab. Co. , Ltd., 2020) |
|  | Reverse | MiFish-Ayu&Wakasagi | GTGACTGGAGTTCAGACGTGTGCTCTTCCGATCTCATAGTGGGGTATCTAATCCCAGTTTG |  |
| 2nd | Forward | 2nd F | AATGATACGGCGACCACCGAGATCTACAC-Index-ACACTCTTTCCCTACACGACGC | (Miya et al., 2015) |
|  | Reverse | 2nd R | CAAGCAGAAGACGGCATACGAGAT-Index-GTGACTGGAGTTCAGACGTGTG |  |

^1^Each primer used for 1^st^ PCR was mixed the following ratio to make a primer mix; MiFish-U-F/R: MiFish-E-F/R: MiFish-U2-F/R: MiFish-Ayu&Wakasagi = 2: 4: 2: 1.

Table S4. Quantitative real-time PCR (qPCR) for each target species with different preservation methods. Environmental DNA (eDNA) concentration was assessed using qPCR

| **Exp** | **Method** | **BAC** | **ID** | **Target species** | | |
| --- | --- | --- | --- | --- | --- | --- |
|  |  |  |  | ***Acanthopagrus schlegelii*** | ***Cyprinus carpio L.*** | ***Misgurnus anguillicaudatus*** |
|  |  |  |  | **eDNA concentration (copies/ μ L)** | **eDNA concentration (copies/ μ L)** | **eDNA concentration (copies/ μ L)** |
| 1 | Glass filter | - | 1 |  | 11 |  |
|  |  | - | 2 |  | 12.5 |  |
|  |  | - | 3 |  | 7 |  |
|  |  | - | 4 |  | 9.5 |  |
|  |  | + | 1 |  | 8 |  |
|  |  | + | 2 |  | 16 |  |
|  |  | + | 3 |  | 6 |  |
|  |  | + | 4 |  | 6 |  |
|  | Sterivex | - | 1 |  | 53 |  |
|  |  | - | 2 |  | 53.5 |  |
|  |  | - | 3 |  | 47.5 |  |
|  |  | - | 4 |  | 51.5 |  |
|  |  | + | 1 |  | 31.5 |  |
|  |  | + | 2 |  | 38 |  |
|  |  | + | 3 |  | 38.5 |  |
|  |  | + | 4 |  | 31 |  |
|  | QuickConc | - | 1 |  | 64.5 |  |
|  |  | - | 2 |  | 59.5 |  |
|  |  | - | 3 |  | 68 |  |
|  |  | - | 4 |  | 51.5 |  |
|  |  | + | 1 |  | 100 |  |
|  |  | + | 2 |  | 72 |  |
|  |  | + | 3 |  | 101.5 |  |
|  |  | + | 4 |  | 79 |  |
| 2 | Glass filter | + | 1 | 212 | 807 | 24 |
|  |  | + | 2 | 765 | 698 | 25 |
|  |  | + | 3 | 257 | 574 | 19 |
|  |  | + | 4 | 820 | 1,020 | 26 |
|  | Sterivex | + | 1 | 163 | 1,371 | 16 |
|  |  | + | 2 | 109 | 1,632 | 29 |
|  |  | + | 3 | 117 | 1,050 | 39 |
|  |  | + | 4 | 196 | 1,271 | 13 |
|  | QuickConc | + | 1 | 613 | 8,934 | 156 |
|  |  | + | 2 | 1,461 | 8,788 | 299 |
|  |  | + | 3 | 1,366 | 10,513 | 326 |
|  |  | + | 4 | 1,218 | 11,305 | 292 |

Table S5. Detailed list of fish species detected from different concentration methods by eDNA metabarcoding

1. River water

| Species name | Glass Filter | | | | Sterivex | | | | QuickConc | | | |
| --- | --- | --- | --- | --- | --- | --- | --- | --- | --- | --- | --- | --- |
|  | 1 | 2 | 3 | 4 | 1 | 2 | 3 | 4 | 1 | 2 | 3 | 4 |
| Total read count | 171,252 | 169,237 | 183,750 | 191,255 | 162,975 | 165,998 | 102,986 | 108,245 | 108,150 | 153,412 | 120,697 | 143,781 |
| Total read count (after assignment to species) | 95,374 | 73,745 | 90,310 | 34,769 | 65,571 | 59,992 | 69,339 | 59,337 | 84,102 | 85,094 | 66,286 | 69,448 |
| *Anguilla japonica* | 766 | 0 | 0 | 0 | 0 | 0 | 0 | 0 | 0 | 267 | 0 | 0 |
| *Carassius cuvieri* | 0 | 0 | 0 | 164 | 0 | 0 | 559 | 0 | 0 | 0 | 0 | 0 |
| *Carassius* sp. | 1,760 | 0 | 1,681 | 0 | 465 | 506 | 674 | 867 | 634 | 444 | 223 | 236 |
| *Cyprinus carpio* | 88,320 | 69,625 | 85,932 | 31,643 | 57,752 | 54,927 | 60,447 | 54,677 | 80,441 | 79,735 | 63,941 | 66,208 |
| *Gnathopogon* sp. | 0 | 0 | 0 | 0 | 385 | 226 | 1,342 | 0 | 0 | 708 | 11 | 276 |
| *Hemibarbus* sp. | 0 | 0 | 0 | 500 | 0 | 88 | 0 | 0 | 118 | 0 | 0 | 0 |
| *Lepomis macrochirus* | 0 | 0 | 0 | 0 | 0 | 0 | 0 | 0 | 0 | 0 | 78 | 332 |
| *Liobagrus reinii* | 0 | 0 | 0 | 110 | 0 | 0 | 0 | 0 | 3 | 10 | 35 | 0 |
| *Micropterus nigricans* | 0 | 0 | 0 | 0 | 0 | 0 | 0 | 413 | 0 | 0 | 0 | 0 |
| *Misgurnus anguillicaudatus* | 0 | 446 | 0 | 0 | 203 | 395 | 0 | 0 | 77 | 0 | 0 | 0 |
| *Mugil cephalus* | 6 | 0 | 0 | 0 | 0 | 0 | 0 | 2 | 0 | 0 | 0 | 0 |
| *Nipponocypris temminckii* | 0 | 0 | 0 | 0 | 0 | 186 | 472 | 0 | 20 | 0 | 0 | 0 |
| *Odontobutis obscura* | 0 | 557 | 0 | 13 | 448 | 0 | 0 | 0 | 250 | 315 | 208 | 425 |
| *Oryzias latipes* | 0 | 0 | 0 | 86 | 0 | 0 | 0 | 0 | 229 | 0 | 0 | 0 |
| *Plecoglossus altivelis* | 0 | 0 | 0 | 0 | 277 | 0 | 0 | 0 | 0 | 92 | 0 | 77 |
| *Pseudogobio agathonectris* | 0 | 0 | 0 | 0 | 0 | 0 | 0 | 0 | 0 | 67 | 0 | 0 |
| *Pseudogobio esocinus* | 0 | 0 | 0 | 0 | 562 | 0 | 0 | 0 | 0 | 81 | 261 | 80 |
| *Pungtungia herzi* | 0 | 0 | 0 | 0 | 0 | 0 | 386 | 0 | 0 | 0 | 97 | 0 |
| *Rhinogobius flumineus* | 0 | 0 | 0 | 407 | 0 | 411 | 1,013 | 591 | 354 | 311 | 88 | 400 |
| *Sarcocheilichthys* sp. | 0 | 0 | 0 | 0 | 0 | 0 | 0 | 0 | 0 | 0 | 72 | 0 |
| *Silurus* sp. | 0 | 0 | 0 | 0 | 37 | 63 | 0 | 303 | 6 | 16 | 88 | 71 |
| *Squalidus* sp. | 1,349 | 0 | 0 | 252 | 460 | 565 | 0 | 202 | 120 | 220 | 171 | 0 |
| *Tachysurus nudiceps* | 0 | 35 | 840 | 0 | 410 | 38 | 753 | 165 | 64 | 51 | 200 | 14 |
| *Zacco platypus* | 3,173 | 3,082 | 1,857 | 1,594 | 4,572 | 2,587 | 3,693 | 2,117 | 1,786 | 2,777 | 813 | 1,329 |

1. Sea water

| Species name | Glass filter | | | | Sterivex | | | | QuickConc | | | |
| --- | --- | --- | --- | --- | --- | --- | --- | --- | --- | --- | --- | --- |
|  | 1 | 2 | 3 | 4 | 1 | 2 | 3 | 4 | 1 | 2 | 3 | 4 |
| Total read count | 262,904 | 180,835 | 143,856 | 308,521 | 147,433 | 300,483 | 177,404 | 377,417 | 350,966 | 364,585 | 342,751 | 315,790 |
| Total read count (after assignment to species) | 91,395 | 42,786 | 39,404 | 132,092 | 63,819 | 115,794 | 28,109 | 69,457 | 102,892 | 83,892 | 85,500 | 126,150 |
| *Acanthopagrus latus* | 0 | 0 | 0 | 0 | 0 | 0 | 0 | 2208 | 0 | 0 | 0 | 0 |
| *Acanthopagrus schlegelii* | 41073 | 4754 | 0 | 112510 | 5896 | 15623 | 3 | 6420 | 21807 | 27823 | 9826 | 7677 |
| *Callionymus valenciennei* | 0 | 0 | 0 | 0 | 0 | 0 | 3088 | 0 | 0 | 0 | 0 | 0 |
| *Ditrema* sp. | 0 | 8173 | 0 | 0 | 0 | 0 | 0 | 0 | 0 | 0 | 11144 | 0 |
| *Engraulis japonicus* | 7789 | 8143 | 15019 | 0 | 21704 | 17279 | 0 | 11119 | 0 | 7991 | 11613 | 19164 |
| *Hyporhamphus sajori* | 0 | 0 | 0 | 2661 | 0 | 2473 | 0 | 5359 | 23721 | 0 | 0 | 0 |
| *Konosirus punctatus* | 0 | 0 | 0 | 8 | 0 | 11317 | 0 | 0 | 0 | 0 | 0 | 9683 |
| *Lateolabrax japonicus* | 4197 | 0 | 14857 | 0 | 0 | 3881 | 0 | 0 | 0 | 1097 | 0 | 0 |
| *Mugil_cephalus* | 37255 | 14577 | 0 | 13966 | 23705 | 48132 | 15518 | 34896 | 48022 | 39451 | 50854 | 70739 |
| *Sardinella zunasi* | 1072 | 0 | 4847 | 2057 | 0 | 9636 | 7452 | 3416 | 0 | 2033 | 0 | 12910 |
| *Sardinops* sp. | 0 | 0 | 0 | 0 | 0 | 0 | 0 | 2442 | 0 | 0 | 0 | 0 |
| *Sebastiscus marmoratus* | 0 | 0 | 0 | 0 | 0 | 0 | 0 | 1568 | 0 | 0 | 0 | 0 |
| *Seriola quinqueradiata* | 0 | 0 | 0 | 0 | 0 | 0 | 2048 | 0 | 3035 | 0 | 0 | 0 |
| *Pagrus major* | 0 | 0 | 0 | 890 | 4426 | 0 | 0 | 0 | 2382 | 5492 | 2063 | 3013 |
| *Pleuronectidae* spp. | 0 | 0 | 0 | 0 | 3729 | 0 | 0 | 1142 | 0 | 0 | 0 | 0 |
| *Thunnus* sp. | 0 | 0 | 0 | 0 | 0 | 0 | 0 | 0 | 1301 | 0 | 0 | 0 |
| *Trachurus japonicus* | 0 | 1911 | 0 | 0 | 4354 | 664 | 0 | 0 | 0 | 0 | 0 | 0 |
| *Carassius* sp. | 0 | 0 | 0 | 0 | 0 | 0 | 0 | 0 | 1661 | 0 | 0 | 2072 |
| *Cyprinus carpio* | 9 | 0 | 0 | 0 | 5 | 7 | 0 | 716 | 6 | 5 | 0 | 0 |
| *Hemibarbus* sp. | 0 | 0 | 0 | 0 | 0 | 2371 | 0 | 0 | 0 | 0 | 0 | 0 |
| *Hypomesus nipponensis* | 0 | 0 | 0 | 0 | 0 | 0 | 0 | 171 | 0 | 0 | 0 | 0 |
| *Hypophthalmichthys* sp. | 0 | 5228 | 0 | 0 | 0 | 0 | 0 | 0 | 0 | 0 | 0 | 0 |
| *Ictalurus punctatus* | 0 | 0 | 0 | 0 | 0 | 0 | 0 | 0 | 0 | 0 | 0 | 892 |
| *Plecoglossus altivelis* | 0 | 0 | 0 | 0 | 0 | 0 | 0 | 0 | 957 | 0 | 0 | 0 |
| *Pseudogobio esocinus* | 0 | 0 | 4681 | 0 | 0 | 0 | 0 | 0 | 0 | 0 | 0 | 0 |
| *Pungtungia herzi* | 0 | 0 | 0 | 0 | 0 | 4411 | 0 | 0 | 0 | 0 | 0 | 0 |


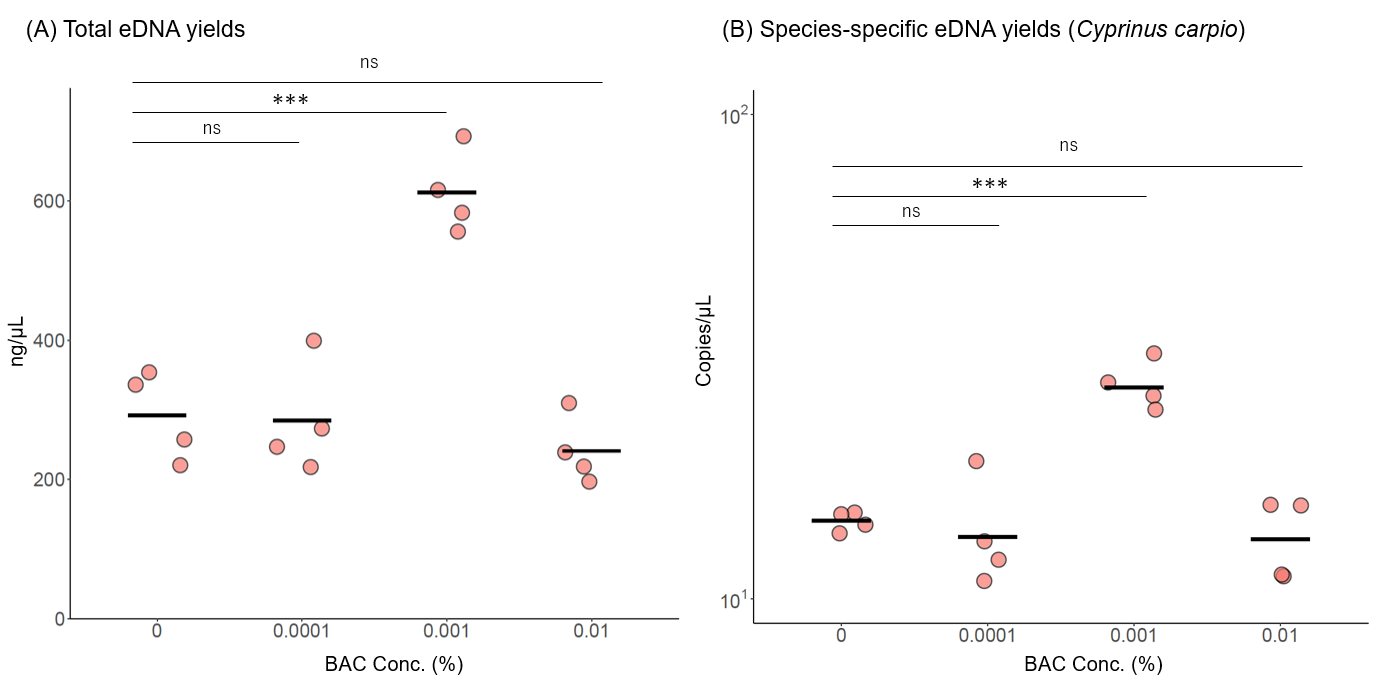


Figure S1. Evaluation of BAC concentration. The experiment was conducted using 400 mL of river water concentrated using QuickConc, compared to the concentration of BAC. BAC was added to the environmental water to a final concentration of 0 %, 0.0001 %, 0.001 % and 0.01 %. For samples with a BAC concentration of 0.01%, excessive foaming occurred during the eDNA concentration process, making it impossible to collect all glass sheets. Therefore, subsequent analyses were conducted using only the recovered glass sheets. Panel (A) shows the total eDNA yields in extracted DNA, and Panel (B) shows the results for *Cyprinus carpio* eDNA recovered from 400 mL of river water using log-scale for the y-axis. Each dot represents a sample; the midlines indicate the averages. Statistical analyses of the differences among groups were performed using Dunnett's test　(*^ns^p* ≧ 0.05, ^***^*p* < 0.001).


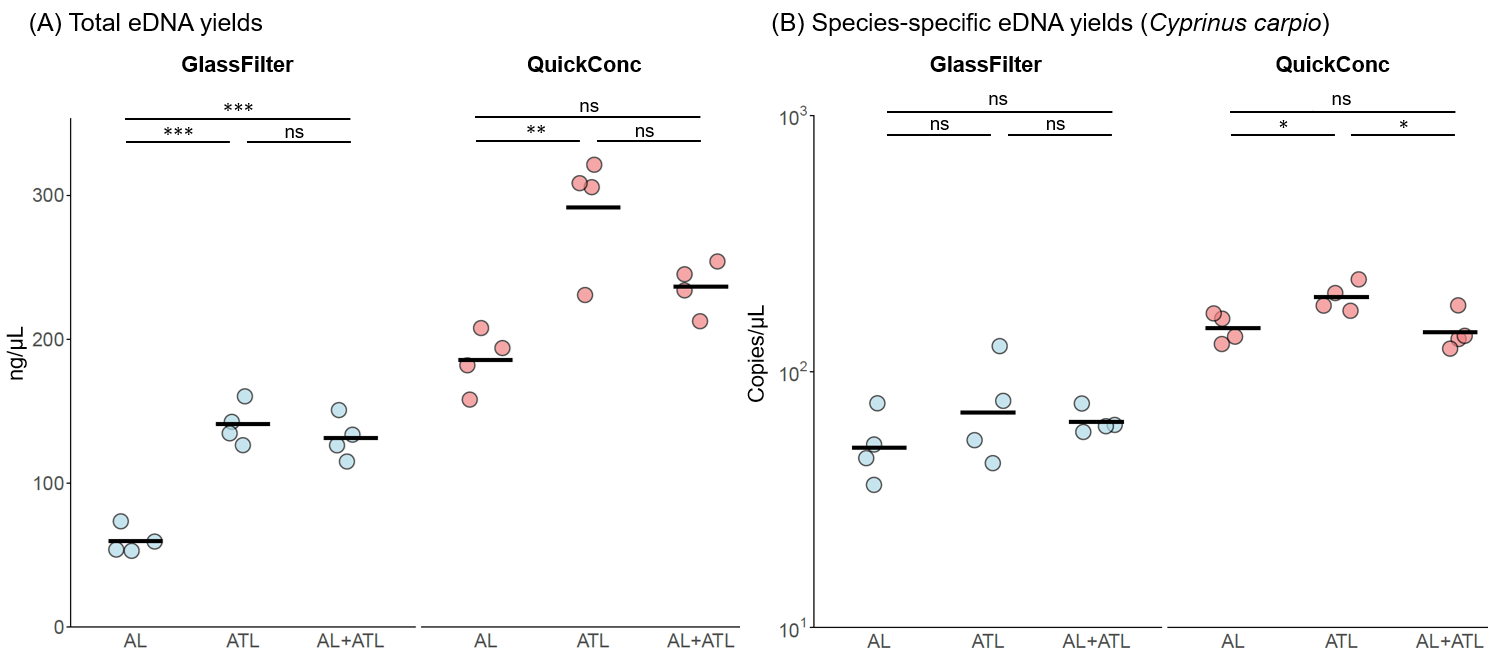


Figure S2. Comparison of the eDNA yields using different extraction buffer. The experiment was conducted using 400 mL of river water concentrated using QuickConc and the glass filter methods. For each method, total eDNA and species-specific eDNA yields were compared under three extraction conditions: using only AL, only ATL, or both AL and ATL for extraction. When both AL and ATL were used, 400 μL of ATL was used during the ProK treatment, and 400 μL of AL was added before ethanol precipitation. Panel (A) shows the total eDNA yields in extracted DNA, and Panel (B) shows the results for *Cyprinus carpio* eDNA recovered from 400 mL of river water using log-scale for the y-axis. Each dot represents a sample; the midlines indicate the averages. Statistical analyses of the differences among groups were performed using Tukey's honest significant difference (HSD) test. The different letters are significantly different factor levels (*^ns^p* ≧ 0.05, ^*^*p* < 0.05, ^**^*p* < 0.01*,* ^***^*p* < 0.001) according to Tukey's HSD.


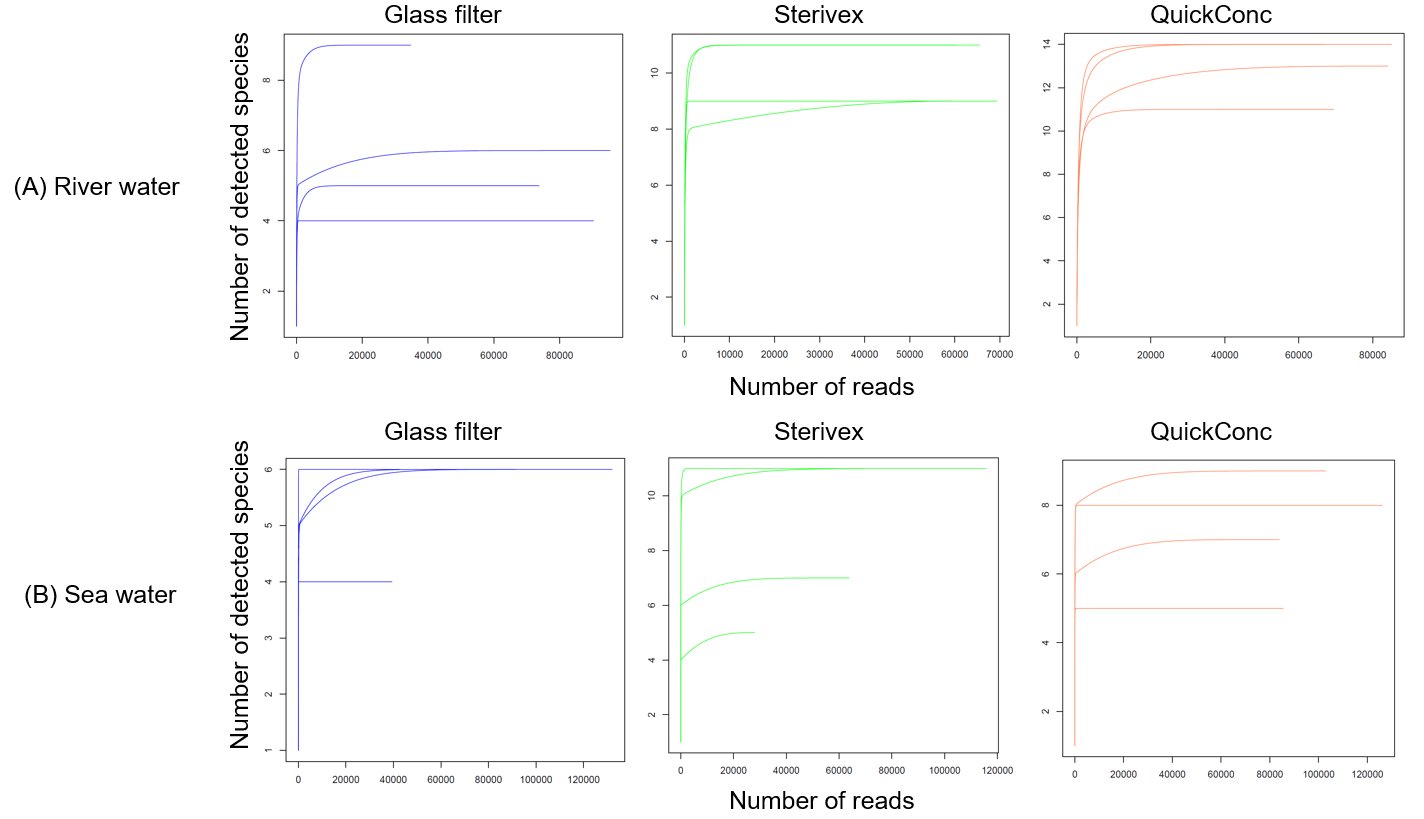


Figure S3. eDNA metabarcoding species accumulation curves. Species accumulation curves for river (A) and sea (B) water show how many species on average are detected with increasing number of reads across three concentration methods: Glass filter (blue), Sterivex (green), and QuickConc (red).


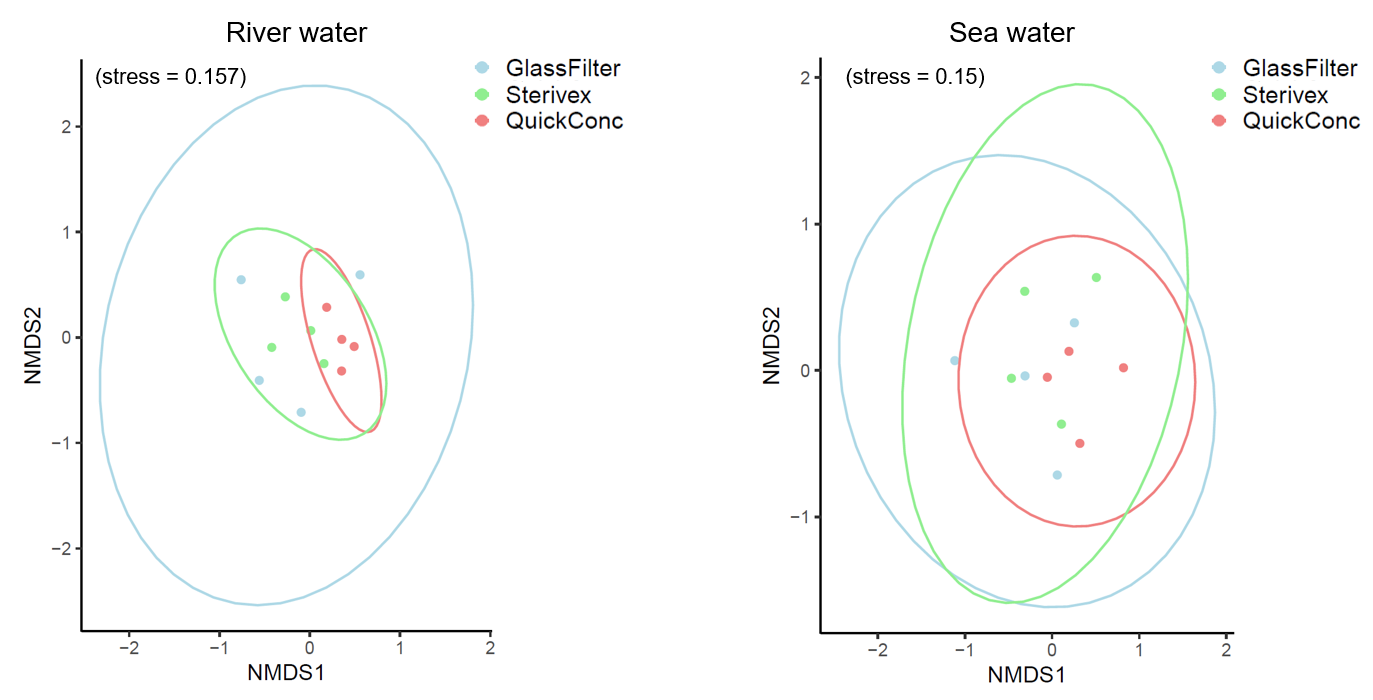


Figure S4. Composition of fish species detected using different preservation methods. A non-metric multidimensional scaling (NMDS) analysis with non-rarefied data was conducted to assess the dissimilarity in estimated fish community composition obtained via different concentration methods. The circle represents confidence intervals around the central tendency of the data points for each method: blue for glass filter, green for Sterivex, and red for QuickConc.


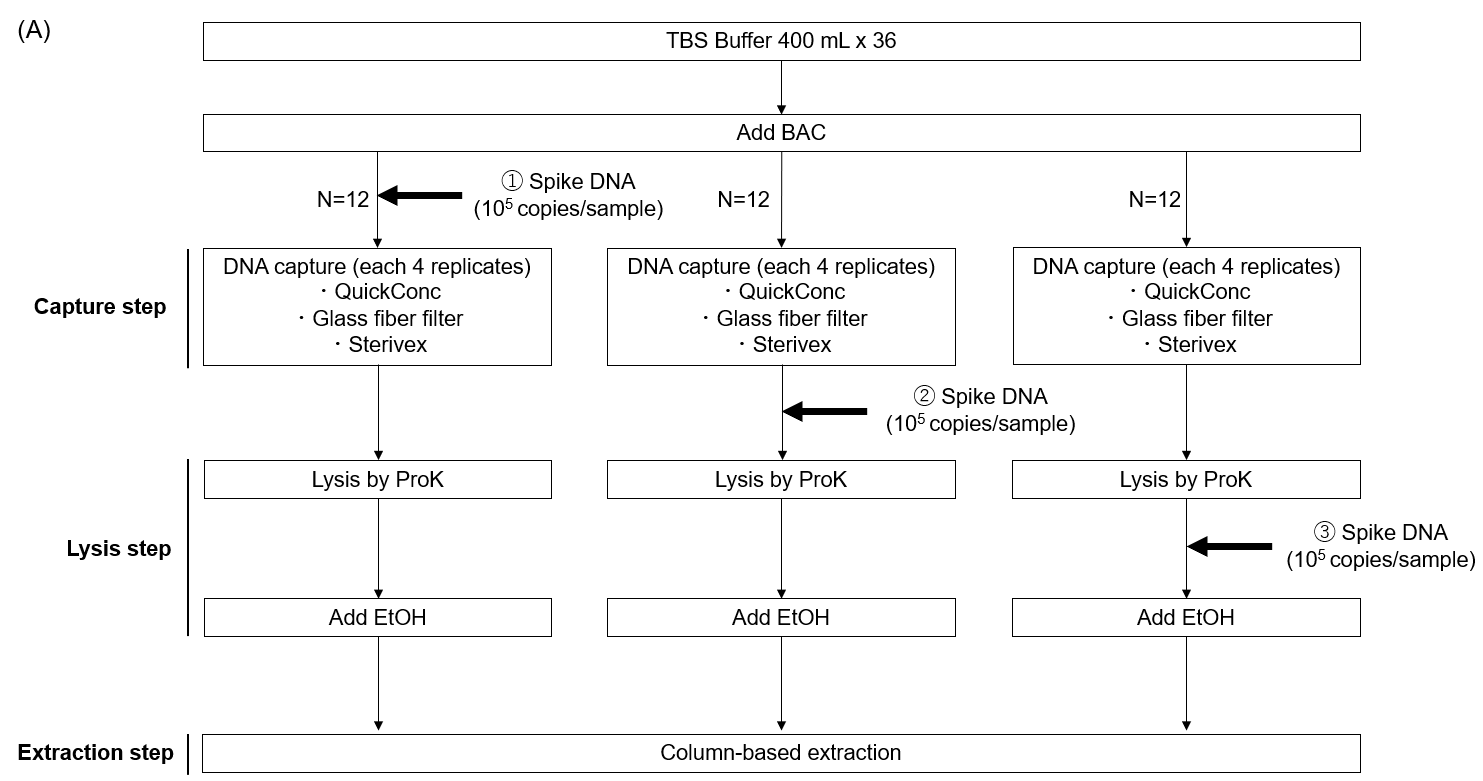


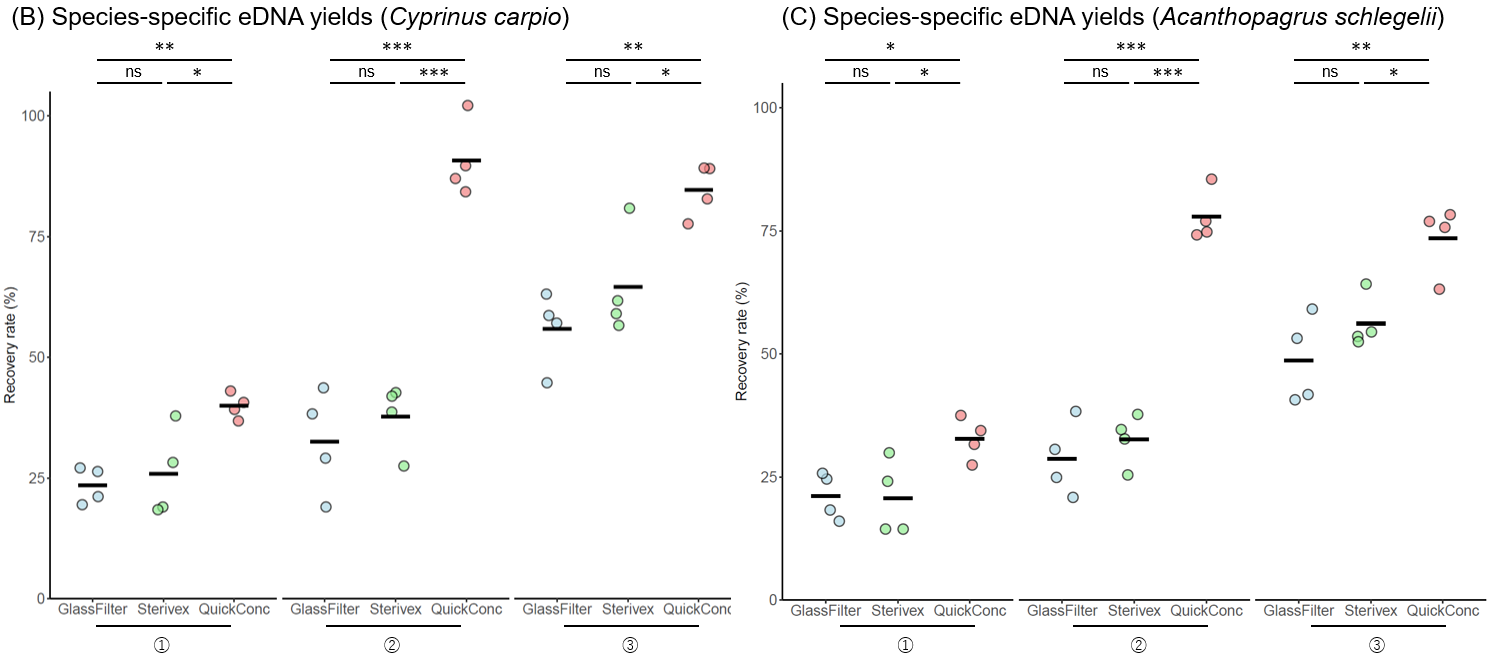


Figure S5 (A). Experiment design diagram of spike-in test. Volumes of 400 mL TBS buffer per sample were used, respectively. Four replicates per condition were conducted, and each was subjected to DNA extraction. Spike DNA consisted of chemically synthesized double-stranded DNA containing sequences for *Cyprinus carpio* and *Acanthopagrus schlegelii*, as detailed in Table S1. The recovery rate of spike DNA was evaluated across three spike-in groups: (1) before concentration, (2) before lysis by ProK, and (3) immediately before addition to the silica column. DNA was added to each group at a concentration of 10^5^ copies per sample.

Figure S5 (B and C). Comparison of the DNA recovery rate. The experiment was conducted using 400 mL of TBS buffer concentrated using QuickConc, compared to the glass filter and Sterivex methods. Panel (B) shows the recovery rate for *Cyprinus carpio* DNA using log-scale for the y-axis, and Panel (C) shows the recovery rate for *Acanthopagrus schlegelii* DNA using log-scale for the y-axis. Each dot represents a sample; the midlines indicate the averages. Statistical analyses of the differences among groups were performed using Tukey's honest significant difference (HSD) test. The different letters are significantly different factor levels (*^ns^p* ≧ 0.05, ^*^*p* < 0.05, ^**^*p* < 0.01*,* ^***^*p* < 0.001) according to Tukey's HSD.
